# Probing Cellular Activity Via Charge‐Sensitive Quantum Nanoprobes

**DOI:** 10.1002/adma.202505107

**Published:** 2026-02-04

**Authors:** Uri Zvi, Shivam Mundhra, David Ovetsky, Qing Chen, Aidan R. Jones, Stella Wang, Maria J. Román‐Vazquez, Marie Kim, Udoka M. Ibeh, Michele Ferro, Kunle Odunsi, Marina C. Garassino, Michael E. Flatté, Melody A. Swartz, Denis R. Candido, Aaron Esser‐Kahn, Peter C. Maurer

**Affiliations:** ^1^ Pritzker School of Molecular Engineering University of Chicago Chicago USA; ^2^ Department of Physics University of Chicago Chicago USA; ^3^ Department of Chemistry University of Chicago Chicago USA; ^4^ Pritzker School of Medicine University of Chicago Chicago USA; ^5^ Section of Hematology/Oncology Department of Medicine The University of Chicago Chicago IL USA; ^6^ University of Chicago Medicine Comprehensive Cancer Center Chicago IL USA; ^7^ Department of Physics and Astronomy University of Iowa Iowa City USA; ^8^ Department of Applied Physics Eindhoven University of Technology Eindhoven The Netherlands; ^9^ Applied Mathematical and Computational Sciences University of Iowa Iowa City Iowa USA; ^10^ Investigator Chan‐Zuckerberg Biohub Chicago LLC Chicago USA; ^11^ Center for Molecular Engineering and Materials Science Division Argonne National Laboratory Lemont IL USA

**Keywords:** band bending, charge transfer, diamond nanocrystals, electric‐field sensing, nitrogen‐vacancy centers, quantum biosensing, zero‐field splitting

## Abstract

Nitrogen‐vacancy (NV) based quantum sensors hold great potential for real‐time single‐cell sensing with far‐reaching applications in fundamental biology and medical diagnostics. Although highly sensitive, the mapping of quantum measurements onto cellular physiological states has remained an exceptional challenge. Here, we introduce a novel quantum sensing modality capable of detecting changes in cellular activity. Our approach is based on the detection of environment‐induced charge depletion within an individual particle that, owing to a previously unaccounted transverse dipole term, induces systematic shifts in the zero‐field splitting (ZFS). Importantly, these charge‐induced shifts serve as a reliable indicator for lipopolysaccharide (LPS)‐mediated inflammatory response in macrophages. Furthermore, we demonstrate that surface modification of our diamond nanoprobes effectively suppresses these environment‐induced ZFS shifts, providing an important tool for differentiating electrostatic shifts caused by the environment from other unrelated effects, such as temperature variations. Notably, this surface modification also leads to significant reductions in particle‐induced toxicity and inflammation. Our findings shed light on systematic drifts and sensitivity limits of NV spectroscopy in a biological environment with ramifications for the critical discussion surrounding single‐cell thermogenesis. Notably, this work establishes the foundation for a novel sensing modality capable of probing complex cellular processes through straightforward physical measurements.

## Graphical abstract

A new quantum sensing modality detects shifts in zero‐field splitting caused by charge rearrangement with diamond nanocrystals in response to cellular activity. These electric‐field‐driven effects provide an alternative to temperature‐based interpretations, enabling real‐time, single‐cell readout of inflammation. This approach reframes NV spectroscopy as a tool for monitoring biologically induced electrochemical changes.

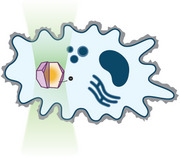

## 1 Introduction

NV centers in diamond nanocrystals enable the probing of physical properties underlying complex biological processes. The remarkable sensitivity of NV centers to magnetic fields has enabled groundbreaking applications, including the detection of subcellular assemblies in magnetotactic bacteria [1], action potentials in individual neurons [2], and mitochondrial activities [3]. Similarly, NV‐based nanothermometry has facilitated the control of thermal gradients in living organisms, offering insights into embryogenesis [4] and introducing novel approaches to neural stimulation [5]. Although promising, these current sensing modalities provide only limited information on cellular activity. Expanding the scope to monitor cellular activities through their chemical environment would unlock new opportunities, offering complementary insights beyond those accessible through magnetic field and temperature sensing. Such an approach could illuminate processes ranging from metabolic activities and cellular stress responses to progression through the cell cycle, apoptosis, differentiation pathways, and immune cell activation.

An important example is the inflammatory activation of macrophages in response to bacterial signals such as LPS, a process central to host defense, tissue remodeling, and many chronic diseases. Conventional methods for tracking this activation include cytokine secretion assays, which average responses across cell populations; transcriptomic approaches such as RNA‐seq, which are inherently destructive and limited to endpoint measurements; flow cytometry, which requires extensive labeling and offers limited temporal resolution; and fluorescent reporters (labels for ROS, NF‐κB, etc.), which often suffer from low robustness due to photobleaching and signal drift [6]. These limitations underscore the need for complementary tools capable of minimally perturbative, real‐time readouts of immune activation at the single‐cell level.

**FIGURE 1:**
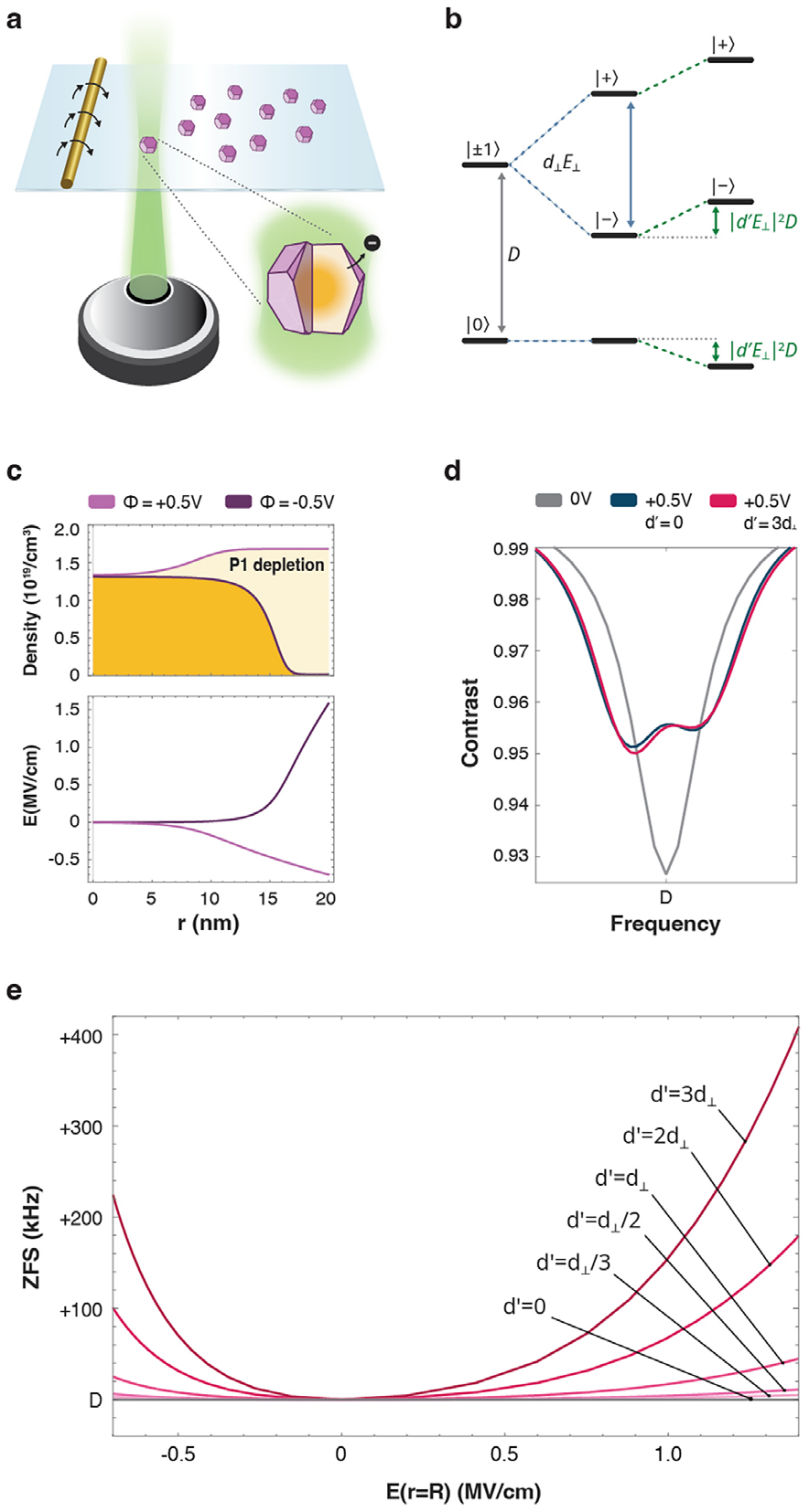
Theoretical framework for sensing electric fields with randomly oriented spins. (a) Illustration of the system's components, including diamond nanocrystals hosting randomly oriented NV ensembles, a Rabi drive, and a laser excitation that result in electron transfer between the diamond and the environment. The depletion of P1 centers from the surface inwards is illustrated by an orange gradient in the nanocrystal's slice. (b) NV energy level diagram illustrating both the effect of the dipole moment $d_{⊥}$ (blue arrow), and the second‐order correction due to the dipole moment $d^{\prime }$ (green arrows). (c) The bare diamond P1 density (upper panel) and the radial electric field profile (lower panel) shaped by a surface potential of −0.5 V (light purple) and +0.5 V. (d) Simulated ODMR curves composed of 100 randomly oriented NVs for no surface potential (gray), as well as for Φ=−0.5 V without ($d^{\prime }=0$, black) and with ($d^{\prime }=3d_{⊥}$, red) the secondary transverse dipole term. (e) The shift in ZFS as a function of electric field at the surface with $d^{\prime }=0,\frac{d_{⊥}}{3},\frac{d_{⊥}}{2},d_{⊥},2d_{⊥}$, and $3d_{⊥}$. The extraction of electric field changes and their corresponding ZFS shifts strongly depends on the exact value of $d^{\prime }$.

Our approach leverages the sensitivity of NV centers in diamond nanoparticles to environment‐induced charge transfer. Each nanoparticle, hosting an ensemble of approximately 100 NV centers (Figure 1a), acts as a nanoscale sensor capable of detecting subtle changes in its surroundings. Recent findings [7, 8, 9, 10] have demonstrated that surface potential‐induced band bending in diamond leads to the ionization of substitutional nitrogen defects (P1 centers), creating a localized charge layer. This charge layer, in turn, generates an electric field that can be precisely measured using NV‐based optically detected magnetic resonance (ODMR) spectroscopy.

In this work, we establish a readout approach designed to probe environment‐induced electric field changes in diamond nanocrystals through measurable shifts in their ODMR spectra away from their initial state. This measurement approach is based on a comprehensive model accounting for the influence of the commonly neglected secondary transverse dipole term ($d^{\prime }$). We demonstrate the significance of our model in saline buffer and show that the observed ZFS shifts are effectively suppressed in diamond nanocrystals encapsulated in biocompatible core‐shell structures — further supporting our hypothesis that these effects result from surface interactions with the environment. Utilizing our newly developed sensing modality, we show that the identified ZFS shifts enable us to differentiate cellular activation states, as exemplified in RAW cells stimulated with LPS. Finally, we discuss the implications of the observed ZFS shift for ODMR‐based sensing, particularly in nanoscale thermometry, and highlight its potential applications in monitoring a broad spectrum of cellular processes.

### 1.1 Sensing with Randomly Oriented Spins

Conventional NV‐based electric field sensing relies on coupling fields to the NV's axial $d_{∥}$ or transverse $d_{⊥}$ dipole moments [11]. Crucially, these interactions depend on the relative orientation of each NV center with respect to the local electric field. For NV ensemble as well as cases in which the sensor is not fixed in space, this orientation dependence results in an inhomogeneous broadening of the ODMR line [12] but not a static overall ZFS shift, severely limiting its utility in biological settings. The absence of any systemic shift makes it challenging to separate the effect of electric field‐induced line broadening from other noise sources, such as magnetic fields.

In our approach, we go beyond this simple model and include the contribution from the transverse dipole term $d^{\prime }$, which is estimated to be of similar magnitude as $d_{⊥}$ yet is typically neglected (Figure 1b) [12, 13, 14]. The full Hamiltonian then takes the form [15, 16]:

(1) $$\begin{array}{ccc} \frac{H}{h} & = & \left(D+d_{∥}E_{z}\right)\left(S_{z}^{2}-\frac{2}{3}\right)+d_{⊥}\left(E_{x}\left(S_{y}^{2}-S_{x}^{2}\right)+E_{y}\left\{S_{x},S_{y}\right\}\right)\\ & & +\;\;d^{\prime }\left(E_{x}\left\{S_{x},S_{z}\right\}+E_{y}\left\{S_{y},S_{z}\right\}\right), \end{array}$$

where $E=(E_{x},E_{y},E_{z})$ denotes the electric field at the location of the NV center, D the temperature‐sensitive part of the ZFS, h the Planck's constant, {·,·} the anticommutator, and $S=(S_{x},S_{y},S_{z})$ the spin‐1 matrices.

Using second‐order perturbation theory and assuming that the ensemble average satisfies $\langle E_{z}\rangle \to 0$, we find that the ODMR frequency shift is determined by $\approx \left\langle |E_{⊥}{|}^{2}\right\rangle |d^{\prime }{|}^{2}/D$, where $E_{⊥}=\sqrt{E_{x}^{2}+E_{y}^{2}}$ represents the transverse field.

We connect the local electric field to the surface potential (ϕ) and the specific diamond doping profile. Following the procedure described in ref. [7], we solve Poisson's equation, accounting for the P1, vacancies, and NVs densities [17, 18] (**Extended Figure E1**). Figure 1c describes the P1 densities (upper panel) and the resulting electric field profiles (lower panel) for an idealized spherical particle, where Φ=0.5 V, accounts for the surface‐potential of a bare oxygen‐terminated diamond [7], and Φ=−0.5 V, the case of a nanocrystal that has undergone a depletion of P1 centers due to an electron transfer from the diamond to the environment.

Finally, using Lindblad formalism (see methods and Supporting Information note S1), we simulate the ODMR spectrum for an average of 100, randomly oriented NV centers under continuous‐wave laser excitation and microwave driving. Our simulations reveal several key properties of this approach. First, the presence of a surface potential Φ=0.5 V vs. Φ=0 V results in a splitting of the resonance into two eigenstates (Figure 1d). Second, the ensemble‐average of the dipole terms results in asymmetric line broadening and contrast changes to the $f^{+}$ and $f^{-}$ transitions (the transitions from |0⟩→|+⟩ and |0⟩→|−⟩, respectively) that are dependent on the relative orientation of each NV to the localized electric field and to the Rabi drive field (Supporting Information note S1). Third, as predicted by our analytical result from Equation 5 (Supporting Information note S1), the addition of $d^{\prime }$ leads to an overall shift in ZFS. A systematic investigation of the ZFS resonance as a function of electric field reveals a quadratic field dependence on $d^{\prime }$ (Figure 1e).

### 1.2 ZFS in Phosphate‐Buffered Saline Environment

**FIGURE 2:**
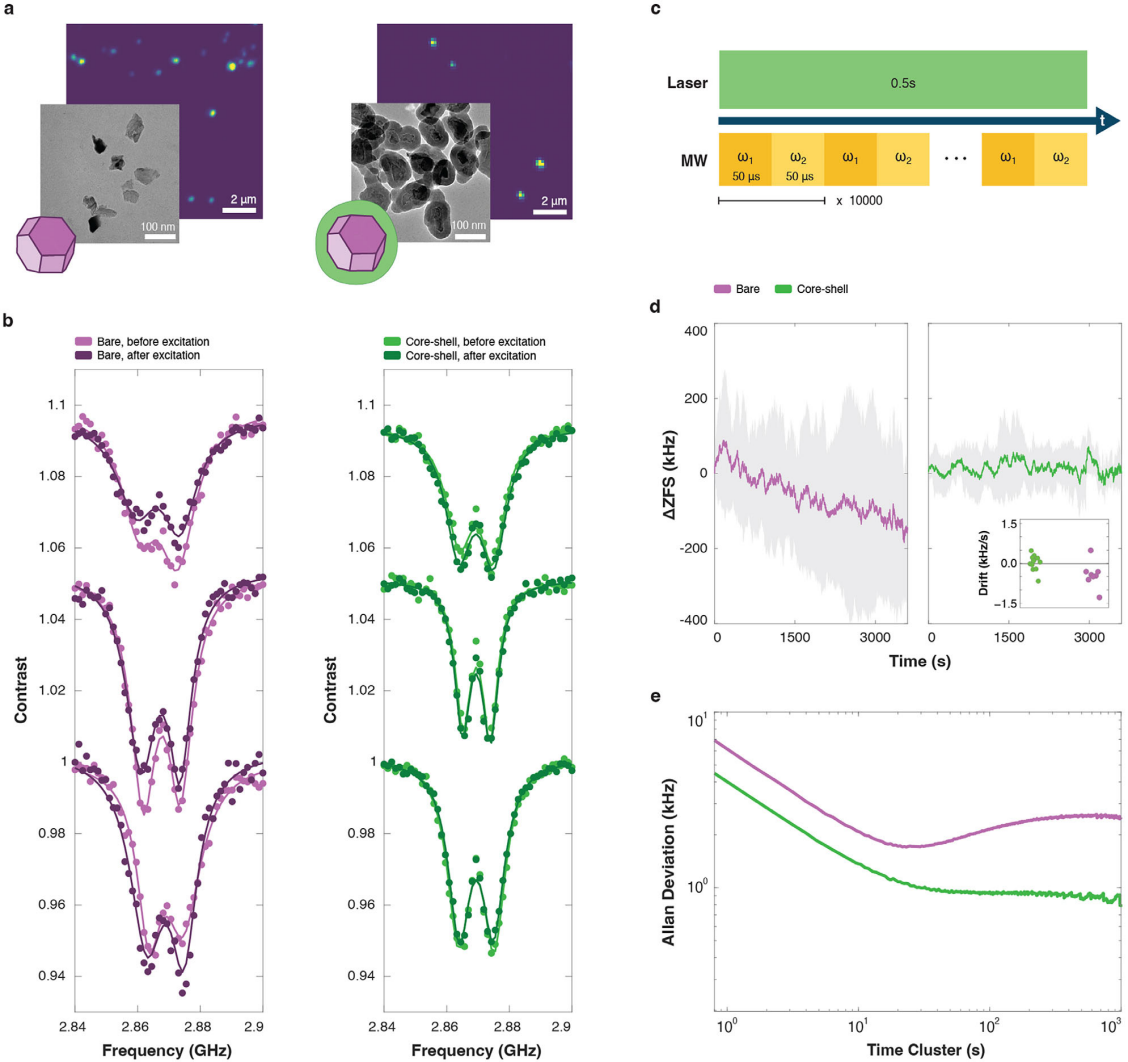
ZFS behavior in PBS. (a) Confocal and transmission electron microscopy (TEM) images of bare (left panel) and core‐shell (right panel) particles. (b) Representative ODMR spectra for bare (left panel) and core‐shell (right panel) particles before (light color) and after (dark color) laser excitation. Points represent experimental data, while lines are double Lorentzian fits. (c) Pulse sequence used to track ZFS with higher temporal resolution. (d) Average ZFS time‐series curves for bare (N=8, left panel) and core‐shell (N=12, right panel) particles during laser excitation. One standard deviation regions are shown in shaded gray areas. Drift terms extracted are shown in the inset for both bare (purple points) and core‐shell (green points) particles. (e) Allan deviation computed from weighted time series curves for bare (purple) and core‐shell (green) particles.

Inspired by our theoretical findings, we investigate whether the properties predicted by our model are apparent in experiments under biologically relevant conditions. We deposited bare diamond nanocrystals with a diameter of 70 nm (Figure 2a, left panel) on a coverslip and investigated the ZFS behavior in phosphate‐buffered saline (PBS), which contains charged ions that approximate the osmolarity and ionic strength of biological fluids. Interestingly, ∼1 hour of laser excitation ($∼0.1\;\mathrm{mW}/\mu m^{2}$) led to significant alterations in the shape of the ODMR spectrum, including asymmetric broadening of the full‐width half maximum (FWHM) and imbalanced changes in the contrast of the $f^{+}$ and $f^{-}$ transitions (**Extended Figure E2** and Figure 2b, left panel). These orientation‐dependent changes are well predicted by our model (Supporting Information notes S1 and S2). In contrast, the ZFS systematically shifted to lower frequencies by an average of −0.45(16) MHz after adding PBS. Such a decrease in the ZFS aligns with a charge transfer and a subsequent reduction in the intra‐particle electric field sensed by the NV centers (Figure 1e). See Supporting Information note S2 for limitations in determining the magnitude of electric field changes. We note that we observed no systematic changes in ZFS when performing these measurements in air and water (**Extended Figure E3**), suggesting that the observed ZFS shifts depend on the chemical environment.

Measuring the ZFS by acquiring a full ODMR spectrum is a time‐consuming process that limits the ability to observe rapid ZFS changes. To capture ZFS dynamics with better temporal resolution, we implemented a two‐point measurement scheme previously developed for NV thermometry [19]. In this scheme, we measure the fluorescence at two microwave frequencies (Figure 2c). An addition of a proportional‐integral‐derivative (PID) control loop and simultaneous spatial tracking (**Extended Figure E4**) enables a rapid and efficient extrapolation of the ZFS value (see Extended Figure E4 and Supporting Information note S3 for a summary of the measurement's performance in constant and modulated temperatures). We used our PID measurement approach to explore the rapid dynamics of the ZFS change in PBS under laser illumination and observed significant temporal drifts (Figure 2d, left panel). In all observed cases, we found that increased illumination time resulted in a larger decrease of the ZFS, suggesting that the observed drifts in ZFS are an optically induced effect.

### 1.3 Reduced Drifts in Core‐Shell Nanoparticles

We hypothesized that exposure of the nanoparticles' surface to PBS is a key factor leading to the observed ZFS shifts. To test this hypothesis, we modified the surface by coating the particles with a 15 nm thick protective silica shell [7]. In contrast to bare diamond nanocrystals in PBS, the ZFS of core‐shell particles remains stable during extended laser excitation, implying a stable surface potential. In fact, we find that the initially measured ODMR spectra (Figure 2b, light green curve) are qualitatively unchanged after continuous laser excitation (Figure 2b), dark green curve. For a time series of the ZFS, see the right panel in Figure 2d. We note that prior to performing the measurements, we applied a short laser pulse in order to initialize a charge equilibrium in the diamond particle (**Extended Figure E5** and Supporting Information note S4). The ZFS drift profile for bare (N=8) and core‐shell (N=12) particles was then quantitatively characterized from their time‐traces using a drift term analysis (methods), revealing negative drift terms for bare particles that significantly differ (see **Extended Data Table E1** for p‐values) from the near zero drift terms of core‐shell particles in PBS (Figure 2d, inset). For additional insight, we employed an Augmented Dickey‐Fuller (ADF) test (See Methods' Statistics section) — a statistical tool to test for non‐stationary processes [20]. A p‐value of 0.557 indicates that the ZFS time series for bare nanocrystals (Figure 2d, left panel) is indeed a non‐stationary process, whereas, for core‐shell particles (Figure 2d, right panel) a p‐value of $3.06\times 10^{-4}$ suggests a stationary process. We further examined the stability of the ZFS by computing the Allan deviation for the time‐domain signal (Figure 2e). This suggests that for short‐time averages (τ<10s) the uncertainty of the ZFS is three times smaller in core‐shell particles compared to their bare counterparts. Furthermore, the Allan deviation for bare particles reveals a systematic drift‐term in the ZFS for times exceeding 20s, whereas for core‐shell particles the Allan deviation plateaus at 0.847 kHz, suggesting that the uncertainty in ZFS has reached a white noise limit.

### 1.4 Effects on Toxicity and Inflammation

While diamond nanocrystals are broadly regarded as biocompatible [21, 22], several studies have reported concentration dependent toxicity [23, 24, 25], particularly in immune cells [26, 27, 28]. To establish concentration ranges suitable for cellular experiments, we evaluated the effects of bare and core‐shell diamond nanoparticles on RAW macrophages, a commonly used murine immune cell line. RAW cells were chosen for their robust inflammatory response characterized by elevated levels of oxidative species and metabolic reprogramming. This response makes them a particularly suitable platform for modulating charge‐transfer events. We first confirmed that both particle types were internalized at comparable levels (**Extended Figure E6(a)**), allowing us to directly compare their cytotoxicity. A confocal study suggested that the vast majority of both bare and core‐shell particles did not reside in lysosomes (Extended Figure E6(b– d)), as supported by previous reports [27, 29, 30, 31].

**FIGURE 3:**
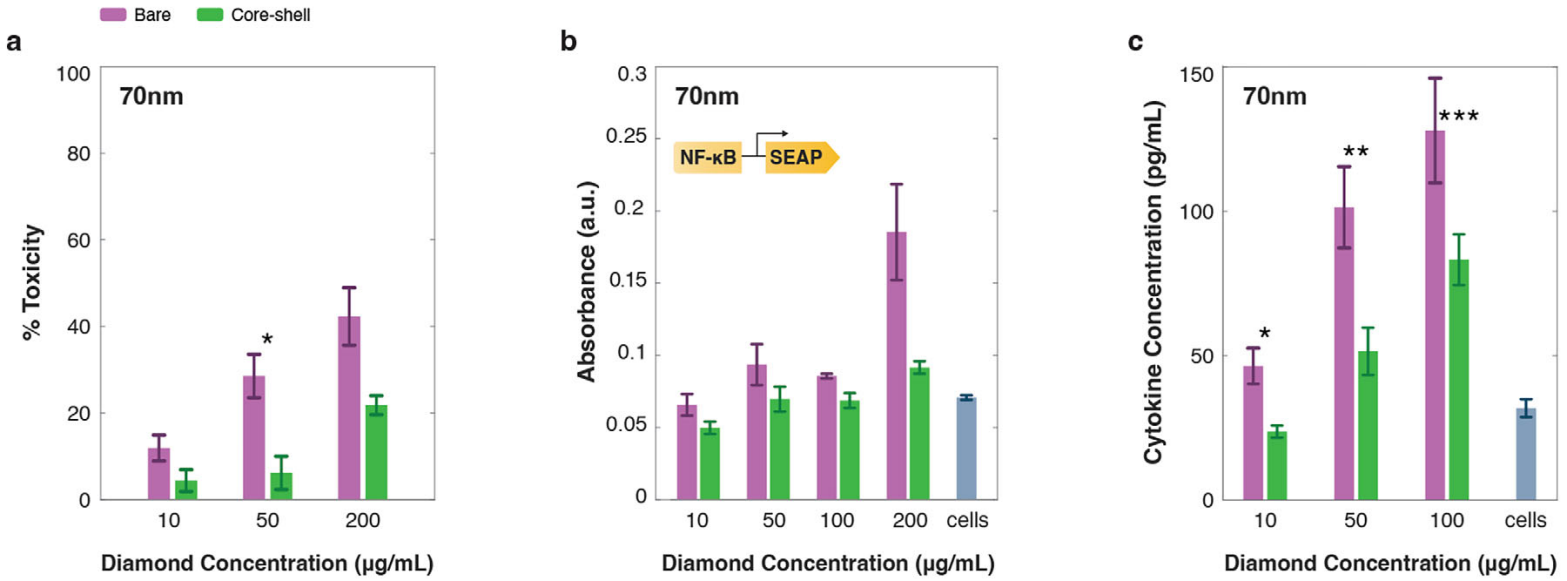
Toxicity and inflammation. (a) % toxicity measured with an LDH secretion assay for cells incubated with different diamond core concentrations of 70 nm bare (purple) and core‐shell (green) particles. (b) NF‐κB expression measured via Quanti‐Blue assay for cells incubated with different diamond core concentrations of 70 nm bare (purple) and core‐shell (green) particles. (c) TNF‐α secretion measured with a LEGENDplex assay for cells incubated with different diamond core concentrations of 70 nm bare (purple) and core‐shell (green) particles. Bright blue groups show measurement for cells with no particles. See methods and Supporting Information note S5 for more information about the protocol. All p‐values are detailed in Extended Data Table E1.

We assessed toxicity by measuring lactate dehydrogenase (LDH) release into the culture medium at 6, 24, and 48 hours following incubation with diamond nanoparticles at concentrations ranging from 10 to 200 μg ${\mathrm{mL}}^{-1}$. Across all time points and concentrations, cells exposed to core‐shell particles released significantly less LDH than those treated with bare nanocrystals (Figure 3a and **Extended Figure E7(a),(d,e)**). To complement the LDH assay, we performed two additional tests at the 24‐hour time point. A live‐dead assay showed no significant difference compared to the control group, except at the highest concentration of bare particles, which showed a slight reduction in cell viability (**Extended Figure E8(a)**). EdU incorporation revealed robust DNA synthesis in cells treated with either bare or core‐shell particles (Extended Figure E8(b)). Interestingly, we observed 15% higher synthesis in cells treated with core‐shell particles at the highest concentration, indicating enhanced proliferative capacity compared to cells treated with bare nanocrystals (see **Extended Figure E9** for live‐dead and EdU gating strategy).

We aimed to measure inflammation by monitoring nuclear factor‐kappa B (NF‐κB) expression levels using a Quanti‐Blue assay. Following overnight incubation, core‐shell particles showed a significant reduction in inflammation at concentrations higher than 50 μg ${\mathrm{mL}}^{-1}$ (Figure 3b) when compared with bare particles. To better understand the inflammatory responses induced by diamond nanocrystals, we analyzed the cytokine release profile. Using a LEGENDplex assay, we measured 13 cytokines and chemokines and observed an inflammatory response solely characterized by elevated levels of TNF‐α (Figure 3c). This pattern is consistent with inflammatory responses seen with other nanoparticle systems [32, 33]. Importantly, while both particle types induced higher levels of TNF‐α secretion at concentrations above 50 μg ${\mathrm{mL}}^{-1}$ compared to control, cells incubated with core‐shell particles exhibited significantly lower TNF‐α levels compared to those exposed to bare particles (see Extended Data Table E1). We repeated the measurements with smaller, 40 nm diameter particles and found that core‐shell particles again reduced the expression of LDH, NF‐κB, and TNF‐α (Extended Figure E7 and Supporting Information note S5). Based on these measurements, we selected 15 μg ${\mathrm{mL}}^{-1}$ as a suitable working concentration for subsequent live‐cell experiments, as it maintained robust cell viability and proliferation while minimizing inflammatory activation.

### 1.5 Detecting Cellular Inflammation Through ZFS Measurement

**FIGURE 4:**
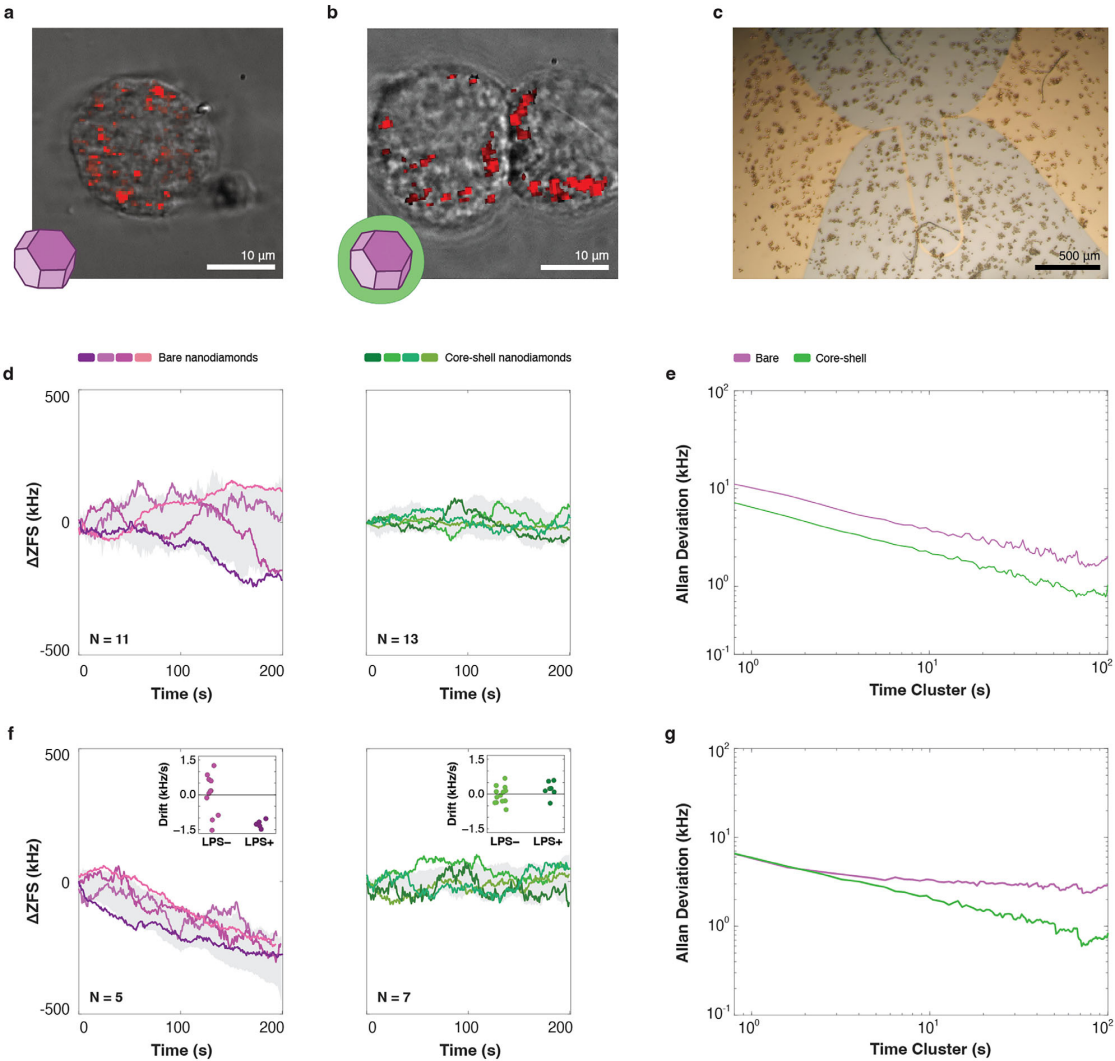
Probing macrophage inflammation with charge sensitive quantum nanoprobes. Cells were imaged following incubation with bare (a) and core‐shell (b) particles. (c) Bright field image of cells growing in a customized well on top of an Ω‐shaped coplanar waveguide (Supporting Information note S8) for ZFS measurements in live cells. (d) ZFS time series curves of four representative bare (purple, left panel, total N=11 particles in 7 different cells) and core‐shell (green, right panel, total N=13 particles in 10 different cells) particles localized in unstimulated RAW cells, and their corresponding Allan deviations in (e). (f) ZFS time series curves of four representative bare (purple, left panel, total N=5 particles in 4 different cells) and core‐shell (green, right panel, total N=7 particles in 4 different cells) particles localized in LPS‐stimulated RAW cells, and their corresponding Allan deviations in (g). Insets in (f) compare drift terms extracted for ZFS tracking of bare (left inset) and core‐shell (right inset) particles in nascent and LPS‐activated cells. Standard deviation calculated from all individual curves is shown as gray shaded areas in the panels within (d) and (f). See Figures E12 and E13 for data from all measured particles.

We conclude our study by demonstrating our sensor's ability to detect cellular activity. As a model system, we focus on the inflammatory response of macrophage‐like RAW cells to the TLR‐agonist LPS. We first incubated cells with bare (Figure 4a) or core‐shell (Figure 4b) particles and investigated the ZFS time‐series with no added stimulation (Figure 4c). The ZFS was tracked over 200 seconds under continuous laser excitation for 11 bare nanocrystals in 7 cells and 13 core‐shell nanoparticles in 9 cells (**Extended Figures E10– 12**). The short measurement time was chosen to minimize phototoxicity and preserve cellular viability. While the investigated diamond nanocrystals exhibited significantly larger ZFS fluctuations compared to those measured in PBS, we did not observe drifts in ZFS for either bare or core‐shell particles (Figure 4d). Notably, bare diamond nanocrystals showed significantly wider variations in their ZFS values compared to core‐shell particles. This trend was also evident in the Allan deviation analysis, which indicated lower variability and greater stability for core‐shell particles (Figure 4e).

To assess ZFS behavior in inflamed cells, we stimulated RAW macrophages with 1.5 μg ${\mathrm{mL}}^{-1}$ of LPS following incubation with bare and core‐shell diamond nanocrystals. Notably, all tested intracellular bare nanocrystals (5 particles in 4 cells) exhibited a systematic ZFS drift over a course of 200 seconds (Figure 4f), with an average total shift of −0.27±0.03 MHz. Such a shift would correspond to a temperature increase of 3.62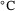 over the course of the measurement, but in our framework, it would instead correspond to a decrease of ∼450 mV in effective surface potential (depending on the value of $d^{\prime }$). To rule out the possibility that observed drifts are a result of LPS‐induced temperature increase, we repeated the measurement on cells incubated with core‐shell particles (7 particles in 5 cells). These measurements showed no sizable ZFS drifts, in strong agreement with our thermodynamical analysis, suggesting that the chemical environment and not temperature is the cause for the observed effects in bare particles (Supporting Information note S6; for temperature sensitivity of bare and core‐shell particles in cells see Extended Figure E10 and Supporting Information note S7). Note, for experimental reasons, ZFS tracking was initiated at various time points, ranging from t=20 to t=100 minutes post‐stimulation, and in all cases continued for 200 seconds per measurement to compare the dynamics with that seen in unstimulated cells. A more careful assessment reveals that all bare diamond nanocrystals in LPS‐activated cells exhibited a negative ZFS drift term (see methods) that significantly differs (*t*‐test p value of $6.38\times 10^{-03}$) from those obtained for unstimulated cells (Figure 4f, left inset). On the other hand, core‐shell particles showed no notable differences compared to unstimulated cells (Figure 4f, right inset). Extended Figures E11 and E12 show individual ΔZFS time traces, which are discussed in Supporting Information note S9. Interestingly, the extracted drift terms in cells were about an order of magnitude larger than those observed in PBS (Figure 2d, inset), indicating a faster shift in electric field. An Allan deviation analysis demonstrates the influence of the drift on measurement sensitivity, with bare particles exhibiting significantly higher deviation at longer cluster times (Figure 4g).

To better understand the molecular nature of the biological process that underlies the ZFS shifts seen in stimulated RAW cells, we tracked the ZFS of bare particles under isolated perturbations. We found that pH (3.5–7), and BSA (300 g $L^{-1}$) did not produce significant ZFS changes (**Extended Figure E13(a,b)**), though incubation with the latter produced small, insignificant changes (∼50 kHz) toward lower frequencies in all but one nanocrystal, which might imply some interaction with the particle surface. In contrast, we found that consistent negative ZFS shifts (mean –296±51 kHz) were produced when bare particles were incubated with a lysate of LPS‐stimulated RAW cells, closely matching our results in live inflamed cells (Figure 4f). Repeating this measurement with core‐shell particles produced no significant ZFS changes (p=0.459). Interestingly, ZFS shifts were again detected when bare particles were exposed to varying $H_{2}O$ 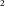 concentration of 1–500 μ m (Extended Figure E13(c,d)). While these shifts were moderate (mean –156±43 kHz) compared to those seen in stimulated live cells and lysate, these preliminary mechanistic studies point toward oxidative stress and lay the groundwork for future investigations. To ensure any signal detected in our cellular measurements is indeed a result of the biological process, and not laser‐induced perturbation, we follow up with phototoxicity and ROS control assays. The results presented in **Extended Figure E14** confirm no laser‐induced oxidative stress in both LPS‐ and LPS+ RAW cells under the illumination conditions used for our quantum measurements (see Supporting Information note S10 for further discussion).

## 2 Discussion and Outlook

We propose that the ZFS shifts reported here are a result of modulations in surface‐potential‐induced band bending that change the charge states of P1 centers in the lattice. Theoretical [17] and experimental [8, 9, 10] studies complement our recent electron paramagnetic resonance (EPR) findings in core‐shell particles [7] and strongly support the susceptibility of P1 centers to surface potential changes. The existence of a charge transfer process is further supported by the increase in ZFS shifts with increased laser power (Extended Figure E5 and Supporting Information note S4), pointing toward the well‐documented photo‐assisted ionization of P1 centers [9, 34]. A steady decrease in PL over the measurements in PBS (Extended Figure E2) suggests that this photoionization might also affect NV centers, while the enhanced PL stability of core‐shell particles points to the suppression of charge transfer between the diamond and its environment. In fact, recent results have shown that such charge transfer can be suppressed in diamond with a dielectric passivation layer [7, 35], as evident in this work by the suppression of ZFS shifts in core‐shell particles.

While the exact mechanism leading to the observed shifts in PBS and stimulated RAW cells merits further investigation, the ZFS shifts toward lower frequencies indicate an overall reduction in the electric field experienced by the NVs. The mitigation of charge‐transfer‐mediated shifts in core‐shell particles allows us to postulate parts of the process. One possibility is that in PBS, ions in the solution may interact with the bare diamond surface, affecting surface potential and charge exchange rates. Additionally, components in the saline solution may change the redox potential near the diamond surface, leading to favorable electron exchange. A depletion of donor P1 centers near the silica‐diamond interface [7], as well as the energy barrier provided by the silica shell, should significantly slow further electron transfer from the ND interior to the environment. Additionally, we recently showed that the silica shell reduces the density of surface defects and other spins [7], which are commonly associated with electron and hole trapping. This trapping phenomenon, widely documented for bulk diamond [36, 37] and bare nanoparticles [38], is greatly suppressed in core‐shell structures. Together, these mechanisms provide evidence for a surface‐induced effect rather than temperature, highlighting our core‐shell particles as an efficient method for decoupling the two effects, thus providing a useful tool for enhanced thermometry in complex biological settings.

In unstimulated RAW cells, we attribute fluctuations in the ZFS to yet‐unidentified processes in the complex cellular environment. The lack of a systematic drift in bare particles is aligned with cellular homeostasis balancing biophysical parameters like oxidative and reducing processes [39, 40] or pH in non‐stimulated cells. Upon LPS stimulation, these immune cells experience a Toll‐like‐receptor (TLR)‐4 dependent inflammatory response that leads to a population shift toward M1 polarization [41]. This shift was confirmed with a polarization analysis (**Extended Figures E15 and E16**), suggesting the cells containing nanocrystals go through the expected associated metabolic reprogramming upon LPS stimulation [42, 43].

Our co‐localization study suggests our sensors are localized in the cytosol (Extended Figure E6), where oxidative shifts [39, 40], up‐ or down‐regulation of active proteins, and ionic fluxes [42] may act individually or synergistically to influence charge transfer between the diamond and the environment. We note, however, that chemical changes due to LPS‐stimulation— such as acidification, redox shifts, and ionic fluxes—are also apparent in endolysosomal and phagosomal compartments [44]. Our investigation ruled out the independent effects of pH and BSA, but confirmed an effect associated with soluble components within the lysate of LPS‐stimulated cells (Extended Figure E13(a– c)). The interaction of these components with the diamond under photo‐excitation likely decreases the magnitude of the negative electric field in the particle by ionization of P1 donors, which in turn leads to the observed reduced ZFS. The detection of larger ZFS shifts with increasing $H_{2}O$ 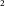 concentrations (Extended Figure E13(d)) suggests that oxidative components play a strong role, but the moderate magnitude of the shifts implies that other components might be crucial to reproduce the strong shifts observed in LPS‐stimulated live cells. Identifying the exact metabolic process and its precise interaction with our diamond sensor is an outstanding question. Decoupling direct reactions with the sensors' surface groups, nanoscale pH changes, and electrochemical potential changes in the environment would require experiments with minimal models to isolate specific influences, an effort we are currently pursuing.

Another interesting direction stems from the reduced toxicity and inflammation of core‐shell compared to bare particles. The reduced toxicity of core‐shell particles is consistent with our previous findings based on other types of silica‐coated particles [32], as well as a growing body of work pointing to the biocompatibility benefits of a passivation layer on bulk [35, 45, 46] and noncrystalline [47, 48, 49, 50] diamond. While both reduced ZFS drift and lower inflammatory response are observed in core–shell particles, no direct mechanistic link is established between the two in this work. Whether the photo‐induced charge exchange between the nanosensors and their surroundings could exacerbate cytotoxic effects, remains an open question for future study.

Our findings also have ramifications for ODMR‐based sensing applications. Our model predicts the asymmetric line broadening and PL contrast of the $f_{\left|0\right\rangle \to \left|+\right\rangle }$ and $f_{\left|0\right\rangle \to \left|-\right\rangle }$ transitions (Figure 1d, Extended Figure E1, and Supporting Information note S2), which have striking implications on the sensitivity limitations of ZFS measurements in cells [4, 51, 52, 53]. More importantly, by considering $d^{\prime }$, we provide a thermodynamically consistent, novel interpretation for the heavily debated systematic ZFS shifts reported in cellular systems [52, 53, 54, 55] that have typically been considered to result from local temperature changes [56].

Ultimately, our study introduces a novel sensing modality that links measurable ZFS shifts to changes in cellular activity. The new understanding of the interplay between the cellular environment and the particle's internal charge profile presents an opportunity for tailoring sensing strategies to monitor complex cellular processes, including cellular differentiation, apoptosis, carcinogenesis, and other metabolic reprogramming events through a simple measurement with quantum sensors. We are currently pursuing such strategies for distinguishing macrophage polarization states, as well as antigen‐specific T‐cell‐induced apoptosis of tumor cells.

## 3 Experimental Section

### 3.1 Theoretical Model from First Principles

Our theoretical ODMR results are simulated using a Lindblad density matrix formalism accounting for the laser excitation, microwave field driving the Rabi oscillations, and all radiative and non‐radiative decays. We have also included relaxation and dephasing processes in the ground state of the NV center. In our formalism, all these processes are described by the Lindblad operators $L_{k}=\sqrt{\Gamma_{ab}}\left|a\rangle \langle b\right|$ with rate $\Gamma_{ab}$ associated to the |b⟩→|a⟩ transition. In the interaction picture, our Lindblad equation for the NV‐j reads

(2) $$\frac{\partial \rho_{j}\left(t\right)}{\partial t}=\frac{i}{\hbar }\left[\rho_{j}\left(t\right),H_{j}\right]+\sum_{k}\left[L_{k}\rho_{j}\left(t\right)L_{k}^{†}-\frac{1}{2}\left\{\rho_{j}\left(t\right),L_{k}^{†}L_{k}\right\}\right],$$

with {A,B}=AB−BA and $H_{j}$ describing the Hamiltonian for the NV‐j. The total photoluminescence of all NVs is obtained via $\mathrm{PL}\left(t\right)=\sum_{j}{\mathrm{Tr}}_{{\mathrm{ES}}_{j}}\left[\rho_{j}\left(t\right)\right]$ where the trace is taken with respect to the excited states (${\mathrm{ES}}_{j}$) of the NVs. The Hamiltonian $H_{j}$ depends on the electric field via Equation (1) , which is calculated for a spherical ND using the solution of the Poisson's equation 

(3) $$\nabla^{2}\phi \left(r\right)=-\rho \left(r\right)/\varepsilon ,$$

with electrostatic potential ϕ(r), charge density ρ(r) and diamond dielectric constant ε. The Poisson equation is numerically solved in a self‐consistent way with charge density accounting for the Nitrogen, vacancy, NV centers, conduction, and valence bands. We solve the equation above with boundary conditions $\phi \left(r=R\right)=\phi_{S}$ and $\frac{d\phi (r=0)}{dr}=0$, with surface electrostatic potential $\phi_{S}$.

For the representative P1 center density, electric field profile, and ODMR curves in Figure 2, ϕ=−0.5 V was calculated using a bare particle with a band‐bending profile described in ref. [7]. However, modeling the non‐spherical, disc‐like shape expected for our diamond nanocrystals [57] as a sphere would lead to an underestimation of the electric field experienced in deeper NVs. Unfortunately, solving Poisson's equation for a disc‐like shape is non‐trivial. Therefore, an effective diameter of 40 nm was chosen for simulating the reported 70 nm particles in this work.

### 3.2 Diamond Nanocrystals

Diamond nanocrystals of 70 nm were obtained from Adámas Nanotechnologies Inc. In brief, type 1b microcrystals are manufactured by static high‐pressure, high‐temperature (HPHT) synthesis and contain about 100–200 ppm of substitutional N. These particles are milled, irradiated with 2–3 MeV, and annealed at 850 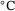 for 2 hours by Adámas Nanotechnologies Inc [18].

### 3.3 Synthesis of Core‐Shell Particles

The growth of uniform silica shells on diamond nanocrystals was performed using a modified silica‐coating technique that utilizes Polyvinylpyrrolidone (PVP) as a stabilizer intermediary to facilitate dense and uniform silica shells formation on nanoparticles [48, 58]. We describe this method in detail in ref. [7]. See Supporting Information note S11 for a description of the exact modifications to coat 70 nm particles.

### 3.4 TEM Characterization

Bare and core‐shell diamond nanocrystals were deposited on a copper (with a formvar carbon film) or Silicon (Si3N4 film) grid while ensuring minimal aggregation, as described in ref. [59]. Images were taken using an FEI Tecnai G2 F30 300kV TEM.

### 3.5 RAW‐Blue Cells

RAW‐Blue Cells(InvivoGen), an NF‐κB‐SEAP reporter Cell line derived from the murine RAW 264.7 macrophages, were cultured (Supporting Information note S12) in T75 flasks (Thermo Fisher). For testing, cells were harvested by scraping and resuspended in 96‐well plates for toxicity and inflammation assays, or in a custom‐made printed circuit board (PCB) design with a coverslip customized for ZFS measurements (Figure 4c and Supporting Information note S8). Cells were allowed to adhere for 6–12 hours before incubation with bare or core‐shell particles.

### 3.6 Measuring ZFS

ODMR spectra were taken using a standard continuous‐wave ODMR sequence with 100 μ sec microwave (MW) pulse duration at an output power of 10–20 dBm from the amplifier. The ZFS was defined as $\frac{f^{+}+f^{-}}{2}$, where $f^{+}$ and $f^{-}$ were obtained from a double Lorentzian fit (Supporting Information note S2). Rapid ZFS measurements were done using an adapted 2‐point measurement [19], with an added PID controller and a particle tracking algorithm (Extended Figure E4 and Supporting Information note S3). In brief, to estimate the ZFS of the NV center over time, given a starting center frequency $\omega_{c}$, we measure the fluorescence at two microwave frequencies, $\omega_{1}=\omega_{c}-\frac{\gamma }{2}$ and $\omega_{2}=\omega_{c}+\frac{\gamma }{2}$, where γ is the full‐width half maximum of the ODMR spectrum of the NV. The fluorescence values at these frequencies, $I_{1}$ and $I_{2}$, are in the quasi‐linear regime of the ODMR Lorentzian spectrum so that changes in their values correspond linearly with changes in the ZFS. As such, measuring $I_{1}$ and $I_{2}$ repeatedly allows us to form updated predictions for the new ZFS. We implement sideband modulation to enable rapid switching of the microwave output between $\omega_{1}$ and $\omega_{2}$ and feed the predicted ZFS value through a proportional‐integral‐derivative (PID) control loop to mitigate noise and fluctuations in the readings (Supporting Information note S3). Running this process continuously over our measurement, we generate estimated ZFS values over time.

### 3.7 ZFS Measurements in Air, Water, and PBS

A drop of NDs (50 μg ${\mathrm{mL}}^{-1}$ in water) was deposited on a plasma‐treated No. 1.5 glass coverslip and shaken for 10 minutes at 100 rpm in a humid chamber to prevent evaporation. After water removal, the coverslip was mounted on a custom PCB, and a coated 25 μm wire was connected across as an RF antenna. A channel (Ibidi bottomless 6 channel slide) was attached to allow delivery and exchange of liquid using a syringe pump (InfusionONE) while the sample was mounted on the microscope stage. ODMR spectra and ∼60 min ZFS tracking data were obtained for bare and core‐shell particles in air ($N_{bare}=8$ and $N_{core-shell}=8$), water ($N_{bare}=7$ and $N_{core-shell}=4$), and PBS ($N_{bare}=9$ and $N_{core-shell}=12$). Measurements were collected across several days under identical conditions and pooled for averaging and statistical analysis.

### 3.8 Cellular Uptake Quantification

Cells incubated overnight with 15 $\mu g\;{\mathrm{mL}}^{-1}$ bare (n=23) or core‐shell (n=17) particles were imaged using our custom‐built microscope (Supporting Information note S13). Fluorescence and bright field images were analyzed using ImageJ [60] to extract integrated fluorescent intensity (total PL intensity) and integrated fluorescent area (total PL area) from internalized particles in each cell (see Supporting Information note S14). The number of measurements, n, represents the number of individual cells measured in each group. Measurements were collected across several days under identical conditions and in parallel to ZFS measurements in cells. Results for each group were pooled for averaging and statistical analysis.

### 3.9 Lysosomal Co‐Localization Analysis

RAW cells were seeded in a μ‐Slide 8 Well high Glass Bottom (ibidi, Cat# 80807) and allowed to adhere overnight before incubation with 15 μg/mL bare or core‐shell particles for 0.5, 1, 2, and 24 hrs. Following incubation, cells in each well were fixed, permeabilized, and stained for lysosomal co‐localization confocal imaging (see details in Supporting Information note S15).

### 3.10 Toxicity and Inflammation Assays

For all toxicity and inflammation assays, RAW‐Blue cells were seeded in 96‐well plates at 12 000 cells per well and incubated overnight at 37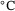 with 5% of ${\mathrm{CO}}_{2}$. Results were collected from triplicate wells analyzed within the same day.

#### LDH Assay

To quantify LDH release, CyQUANT LDH Cytotoxicity Assay (Invitrogen Cat. Nos. C20300) was performed. Cells were incubated in triplicates with 10, 50, and 200 $\mu g\;{\mathrm{mL}}^{-1}$ of bare or core‐shell particles for 6, 24, and 48 hours, before 50 μL of supernatant was transferred from each sample to flat‐bottomed 96‐well plates for colorimetric measurements (Supporting Information note S5).

#### EdU and Live‐Dead Assays

Cells were incubated in triplicates with 10, 50, and 100 $\mu g\;{\mathrm{mL}}^{-1}$ of bare or core‐shell particles for 24 hours. EdU incorporation was assessed using the ClickTech EdU Cell Proliferation Kit (Sigma‐Aldrich, Cat# BCK‐FC488‐50), according to the manufacturer's instructions. Before adding the dye, cells were treated with 10 μM 5‐ethynyl‐2'‐deoxyuridine at 37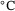 with 5% of ${\mathrm{CO}}_{2}$ for 2 hours.

To quantify cell viability, cells were washed with PBS and stained with Zombie Aqua Fixable Viability Dye (BioLegend, Cat# 423102). The lyophilized dye was reconstituted in 100 μL DMSO, as per the manufacturer's protocol, and diluted 1:500 in PBS immediately before use. Cells were then incubated in the dark with the diluted dye for 15 minutes at room temperature (RT). Following dye staining, cells were washed and fixed with 4% paraformaldehyde (PFA) for 15 min at RT. Permeabilization was performed using 0.1% saponin in PBS for 20 minutes. EdU labeling was performed using the kit's click chemistry reaction cocktail, containing 6‐FAM azide. The cocktail was added to the plates in the dark for 30 minutes at RT before cells were analyzed by flow cytometry (Supporting Information note S16).

#### NF‐κB Assay

To quantify NF‐κB expression, a QUANTI‐Blue assay (InvivoGen Cat. code rep‐qbs) was performed. Cells were incubated in triplicates with 10, 50, 100, and 200 $\mu g\;{\mathrm{mL}}^{-1}$ of bare or core‐shell particles for 16 hours before 20 μL of supernatant was transferred from each sample to flat‐bottomed 96 well plates for colorimetric measurements (Supporting Information note S5).

#### Cytokine Expression Assay

Cytokine expression was measured using a LEGENDplex Mouse Inflammation Panel (BioLegend 740150). Cells were incubated in triplicates with 10, 50, and 100 $\mu g\;{\mathrm{mL}}^{-1}$ of bare or core‐shell particles for 16 hours before 15 μL of supernatant was transferred from each sample to the assay‐provided V‐bottom 96 well plates for addition of Capture Beads, Detection Antibodies, and SA‐PE, followed by flow cytometry (Supporting Information note S5).

### 3.11 ZFS Measurements in Live Cells

Approximately 12 000 RAW‐Blue cells (InvivoGen) were seeded in a well (Ibidi 18 well bottomless μ‐Slide) attached to a coverslip containing a fabricated co‐planar waveguide (Supporting Information note S8). Cells were incubated in media with 15 $\mu g\;{\mathrm{mL}}^{-1}$ of bare or core‐shell particles for ∼6 hours. After incubation, the cells were washed twice, and clear media was added to facilitate imaging. Measurements were done using our custom‐built microscope (Supporting Information note S13), equipped with a live cell imaging chamber (Invivo Scientific, STEV.ECU.HC5 STAGE TOP), and a temperature controller. Since the live‐cell chamber has temperature stability of ±0.5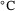 for our sensitive measurement, we added a closed‐loop resistive heater (HT19R), temperature transducer (AD590 in media and HT10KR1 for cells), and controller (TC300) from Thorlabs, which allowed us to achieve temperature stability of <0.1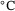. ODMR spectra and ZFS tracking data were collected from 1 to 2 NDs from each cell. Nascent cells were randomly chosen, and NDs were measured immediately (N=11 for bare and N=13 for core‐shell). For inflammation conditions (N=5 for bare and N=7 for core‐shell), cells were stimulated with 1.5 $\mu g\;{\mathrm{mL}}^{-1}$ of LPS and measured at t = 30–90 min post stimulation. In all cases, ZFS tracking was limited to 200 seconds. In addition, all experiments were halted at a total time t≤180 minutes, and cells were monitored using wide‐field microscopy to confirm viability. See Figures E11 and E12 for data from individual particles and assignment to the cell they were measured in.

### 3.12 ZFS in pH, $H_{2}O$, BSA, and Lysate

Diamond nanocrystals and core‐shell particles were deposited onto a glass coverslip as described for ZFS measurements in air, water, and PBS. A well was formed using an Ibidi bottomless μ‐Slide 18 Well, and a liquid exchange system was created using plastic tubing that interfaced with a 1 mL Norm‐ject plastic syringe. ZFS was tracked for bare nanocrystals incubated with varying pH (n=4), varying $H_{2}O$ 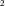 concentration (n=3), 300 g $L^{-1}$ of BSA (n=6), and lysate of LPS‐stimulated RAW cells (n=3). See Supporting Information note S17 for measurement protocols and Extended Figure E13 for results. In all cases, *n* represents the number of individually measured particles. Measurements were collected across several days under identical conditions and pooled for averaging and statistical analysis.

### 3.13 Raw Cells Polarization Assay

For polarization phenotyping, triplicates of cells were incubated with 15 $\mu g\;{\mathrm{mL}}^{-1}$ overnight, washed, and treated with 1.5 $\mu g\;{\mathrm{mL}}^{-1}$ of LPS for ∼60 min. After stimulation, cells were washed and stained with Zombie Aqua Fixable Viability Dye (BioLegend, Cat# 423102), as described in the live‐dead assay protocol. Cells were then washed and fixed with 4% PFA for 15 min at RT and permeabilized with 0.2% (v/v) Tween 20 (PBS) for 10 more minutes. After washing, cells were placed on ice, blocked with anti‐mouse TruStain FcX Plus (BioLegend) for 10 min, and stained with Anti‐CD86 BV605, anti‐TNFa AF647, anti‐CD163 BV421, and anti‐CD206 AF700 (BioLegend) for 15 min. Cells were then washed and analyzed by flow cytometry (Supporting Information note S16). Results were collected from triplicate wells analyzed within the same day.

### 3.14 Phototoxicity and ROS Control Assay

RAW cells were cultured with or without diamond nanocrystals, treated with or without LPS (1.5 $\mu g\;{\mathrm{mL}}^{-1}$, 1 hour), and stained with 5 μ m CellROX green (thermofisher) according to standard protocol. Cells were imaged before and after laser exposure using either 520 nm confocal illumination (200 s per cell, 400 nm diameter laser spot with 8 × 10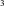 W ${\mathrm{cm}}^{-2}$) or 405 nm wide‐field illumination (45 s, 66 μm diameter illumination spot with 1.5 × 10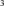 W ${\mathrm{cm}}^{-2}$). Background‐subtracted integrated fluorescence (I) was quantified per cell, and the fractional change was computed as $\left(I_{after}-I_{before}\right)$/$I_{before}$. Unstained replicates were used as negative control. Bright field images were used for qualitative assessment of morphological effects. See Figure E14 Supporting Information note S10 for more information.

### 3.15 Statistics

#### ADF Test

To assess the stationarity of the time series data, we performed the ADF test, which evaluates the presence of a unit root—a key indicator of non‐stationarity. The test equation included a constant and lagged differences to account for autocorrelation, with the optimal lag length determined using the Akaike Information Criterion. A p‐value below 0.05 was considered evidence to reject the null hypothesis, indicating that the series is stationary. See Supporting Information note S18 for more detailed information.

#### Allan Deviation

To compute Allan deviations for individual NV ZFS time‐series data, we recorded the raw fluorescence data for $I_{1}$ and $I_{2}$ and artificially generated data sets for measurements with larger averaging time by summing over subsequent fluorescence data (details in Supporting Information note S18). To compute the average Allan variance plot for the time‐series ZFS data in PBS of bare (n = 8) and core‐shell (n = 12) nanodiamonds in PBS, we applied a weighted average of the individual Allan variance plots, with the weights being the total measurement duration. This method ensures that the aggregate plot accurately reflects the contribution of each time series based on its respective measurement period.

#### Toxicity and Inflammation Bar Plots

All error bars represent standard error unless otherwise noted. All analyses were done using two‐tailed *t*‐tests. See Extended data table E1 for specific p‐values.

#### Intracellular ZFS Measurements

A time series analysis was performed in order to extract drift terms for ZFS tracking data in cells. We first obtained a distribution of differences between consecutive data points and extracted the relevant drift term by calculating the deviation of the mean from zero (Extended Figure E10). The significance between data obtained in nascent and LPS‐activated cells was calculated using a two‐tailed *t*‐test.

## Author Contributions

U.Z., D.R.C, A.E.‐K., and P.M. conceived and designed the study. U.Z., S.M., and D.O. designed and performed the experiments. U.Z., S.M., D.O., A.R.J., and S.W. designed the experimental instruments and software control. D.R.C performed theoretical analysis. D.R.C and M.E.F. provided theoretical model guidance and discussion. U.Z., D.O., and Q.C. designed and performed cellular assays. M.J.R.‐V. guided the LEGENDplex analysis and M.F. contributed toward toxicity and LEGENDplex experiments. U.M.I. and U.Z. performed colocalization experiments. M.K. and Q.C. conducted flow cytometry assays. K.O., M.C.G, M.S., D.R.C., A.E.‐K, and P.M. provided supervision and guidance. U.Z., P.M., D.O., and S.M. wrote the manuscript with extensive input from all authors.

## Conflicts of Interest

The work covered in this manuscript is the subject of a patent application filed by the authors' institutions in the US Patent and Trade Office.

## Supporting information


**Supporting File**: adma72248‐sup‐0001‐SuppMat.pdf.

## Data Availability

The data that support the findings of this study are available from the corresponding authors upon reasonable request.
