# Supplementary material for: Probing Cellular Activity Via Charge‐Sensitive Quantum Nanoprobes
Source: Adv Mater. 2026 Feb 4;38(14):e05107. doi: 10.1002/adma.202505107 (PMC12966981; doi:10.1002/adma.202505107)
Supplement: Supplementary file 1 — Supporting File: adma72248‐sup‐0001‐SuppMat.pdf. [file ADMA-38-e05107-s001.pdf]

Supplementary Material for

Probing cellular activity via charge-sensitive quantum nanoprobes

Uri Zvi[1], Shivam Mundhra[2], David Ovetsky[1], Qing Chen[1], Aidan R. Jones[2], Stella Wang[2], Maria Roman[3], Marie Kim[1], Udoka M. Ibeh[1,4], Michele Ferro[5], Kunle Odunsi[6], Marina C. Garassino[5], Michael E. Flatté[7,8], Melody Swartz[1], Denis R. Candido [†7], Aaron Esser-Kahn [†1,9], and Peter C. Maurer [†1,9,10]

[1]Pritzker School of Molecular Engineering, University of Chicago, Chicago, IL 60637, USA.
[2]Department of Physics, University of Chicago, Chicago, IL 60637, USA.
[3]Department of Chemistry, University of Chicago, Chicago, IL 60637, USA.
[4]Pritzker School of Medicine, University of Chicago, Chicago, IL 60637, USA.
[5]Section of Hematology/Oncology, Department of Medicine, The University of Chicago, Chicago, Illinois, USA.
[6]University of Chicago Medicine Comprehensive Cancer Center, Chicago, IL, USA.
[7]Department of Physics and Astronomy, University of Iowa, Iowa City, Iowa 52242, USA.
[8]Department of Applied Physics, Eindhoven University of Technology, Eindhoven, 5600 MB, The Netherlands.
[9]CZ Biohub Chicago, LLC, Chicago, IL 60642, USA.
[10]Center for Molecular Engineering and Materials Science Division, Argonne National Laboratory, Lemont, IL.
[1†] denis-candido@uiowa.edu, aesserkahn@uchicago.edu, and pmaurer@uchicago.edu

Included in this supplementary Information:

Extended data Figures E1 to E16
Supplementary notes S1 to S18
Supplementary video files V1 and V2
Supplementary references 1 to 27

Extended Data

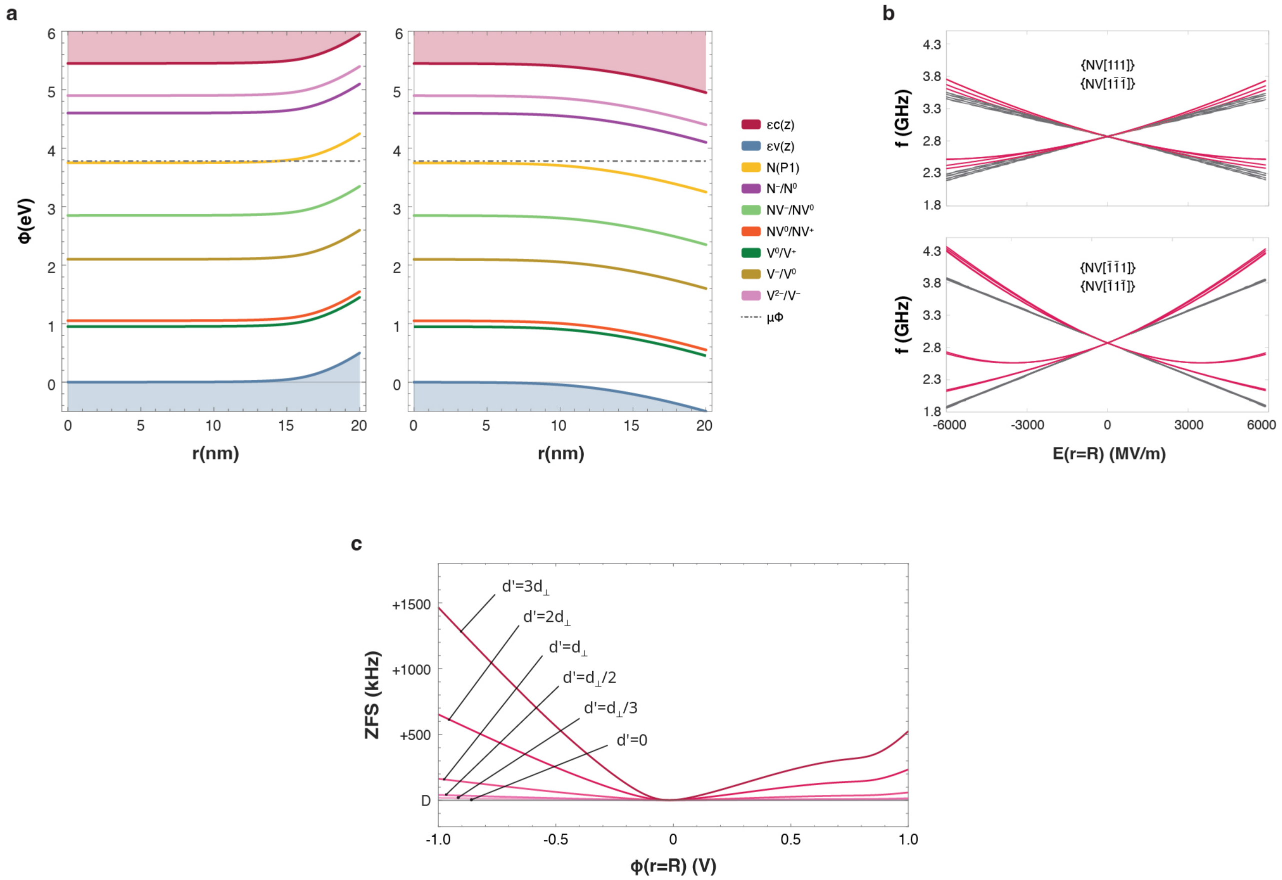

Figure E1: Theoretical model. (a) Band bending simulation of upward (left panel) and downward (right panel) band bending with surface potential of -0.5V and 0.5V, respectively. The downward band bending in carboxylated diamond nanocrystals bends upwards as electrons transfer from the diamond to the environment. (b) $f^{-}\rightarrow f^{+}$ transition frequency for different NV orientations as a function of surface (maximum) electric field for $d' = 0$ (black lines) and $d' = d_{\perp}$ (red lines). The upper panel shows the orientations and anti-orientations of the [111] and the [$1\bar{1}\bar{1}$] NVs. The lower panel shows the orientations and anti-orientations of the [$\bar{1}\bar{1}1$] and the [$\bar{1}1\bar{1}$] NVs. The unidirectional shift in ZFS is apparent for all orientations. Upon a change in the chemical environment (and likely promoted by laser illumination), a charge transfer between the diamond and the environment will change the initial electric field experienced by the NVs. Consequently, a perturbation of the eigenstates of individual NVs leads to an overall shift of the ZFS towards higher or lower frequencies in response to an increase or decrease in the electric field, respectively. (c) Shift in ZFS as a function of surface potential with $d' = 0$, $d_{\perp}/3$, $d_{\perp}/2$, $d_{\perp}$, $2d_{\perp}$, and $3d_{\perp}$.

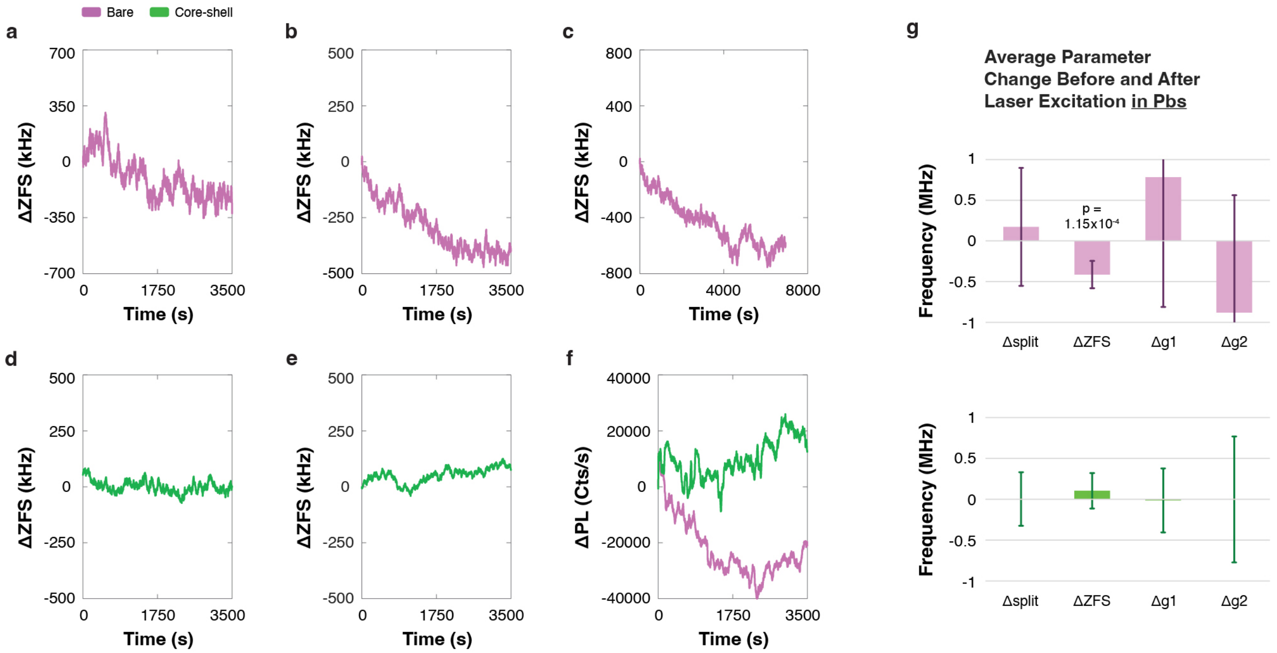

Figure E2: ZFS behavior in PBS. (a-b) Representative ZFS tracking curves during $sim1$ hr of laser excitation of bare particles in PBS. $N = 8$ data sets were averaged to produce the left panel of Figure 2(d). (c) A representative longer time trace of the data showed in (b), demonstrating the continuous drift ZFS for bare particles in PBS. Bare particles did not equilibrate in PBS. (d-e) Representative ZFS tracking curves during $sim1$ hr of laser excitation of core-shell particles in PBS. $N = 12$ data sets were averaged to produce the right panel of Figure 2(d). (f) Change in total PL during ZFS tracking data from (a) and (d), demonstrating the gradual drop in PL in bare particles during excitation, likely due to electron transfer from the diamond to the environment. (g) Distribution of change in key fitting parameters from $N = 9$ bare (upper panel) and $N = 4$ equilibrated core-shell (lower panel) ODMR spectra before and after laser excitation, showing the large variation caused due to divergence from a perfect double Lorentzian spectra (SI note S1 and S2). While the ODMR spectra in Figure 2(b) clearly show a change before and after laser excitation, the only consistent parameter was the change in ZFS.

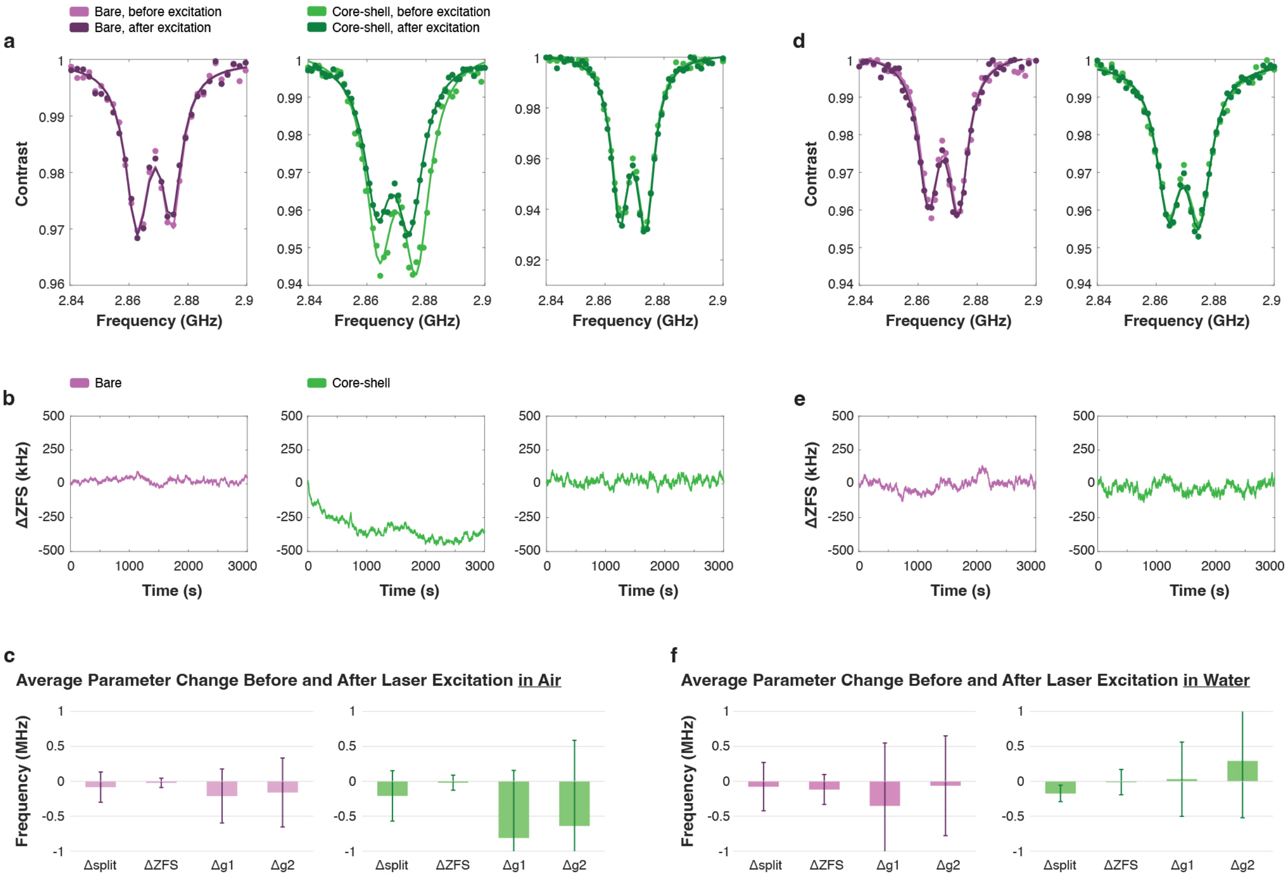

Figure E3: ZFS behavior in air and water. (a) Representative ODMR spectra before (lighter color) and after (darker color) $sim1$ hr of laser excitation in air. Bare particles showed no qualitative difference (left panel), while core-shell particles experienced a left shift towards lower frequencies (middle panel). Following a laser-power-dependent (Extended Figure E5) equilibration, core-shell particles maintained stable spectra (right panel) and no ZFS shifts were detected for several weeks (Extended Figure E5). For all spectra, points represent experimental data, while lines are double Lorentzian fits. (b) Representative ZFS tracking curves during $sim1$ hr of laser excitation in air. The time-series data for bare (left panel), pre-equilibrated core-shell (middle panel), and equilibrated core-shell (right panel) particles confirmed the trends seen in (a). (c) Distribution of change in key fitting parameters from $N = 6$ bare (left panel) and $N = 5$ equilibrated core-shell (right panel) ODMR spectra before and after laser excitation, showing the large variation caused due to divergence from a perfect double Lorentzian spectra (SI note S1 and S2). Note the relatively smaller variations in δZFS. (d) Representative ODMR spectra before (lighter color) and after (darker color) $sim1$ hr of laser excitation of bare (left panel) and core-shell (right panel) particles in MQ-water. (e) Representative ZFS tracking curves during $sim1$ hr of laser excitation of bare (left panel) and core-shell (right panel) particles in MQ-water. (f) Distribution of change in key fitting parameters from $N = 5$ bare (left panel) and $N = 4$ equilibrated core-shell (right panel) ODMR spectra before and after laser excitation.

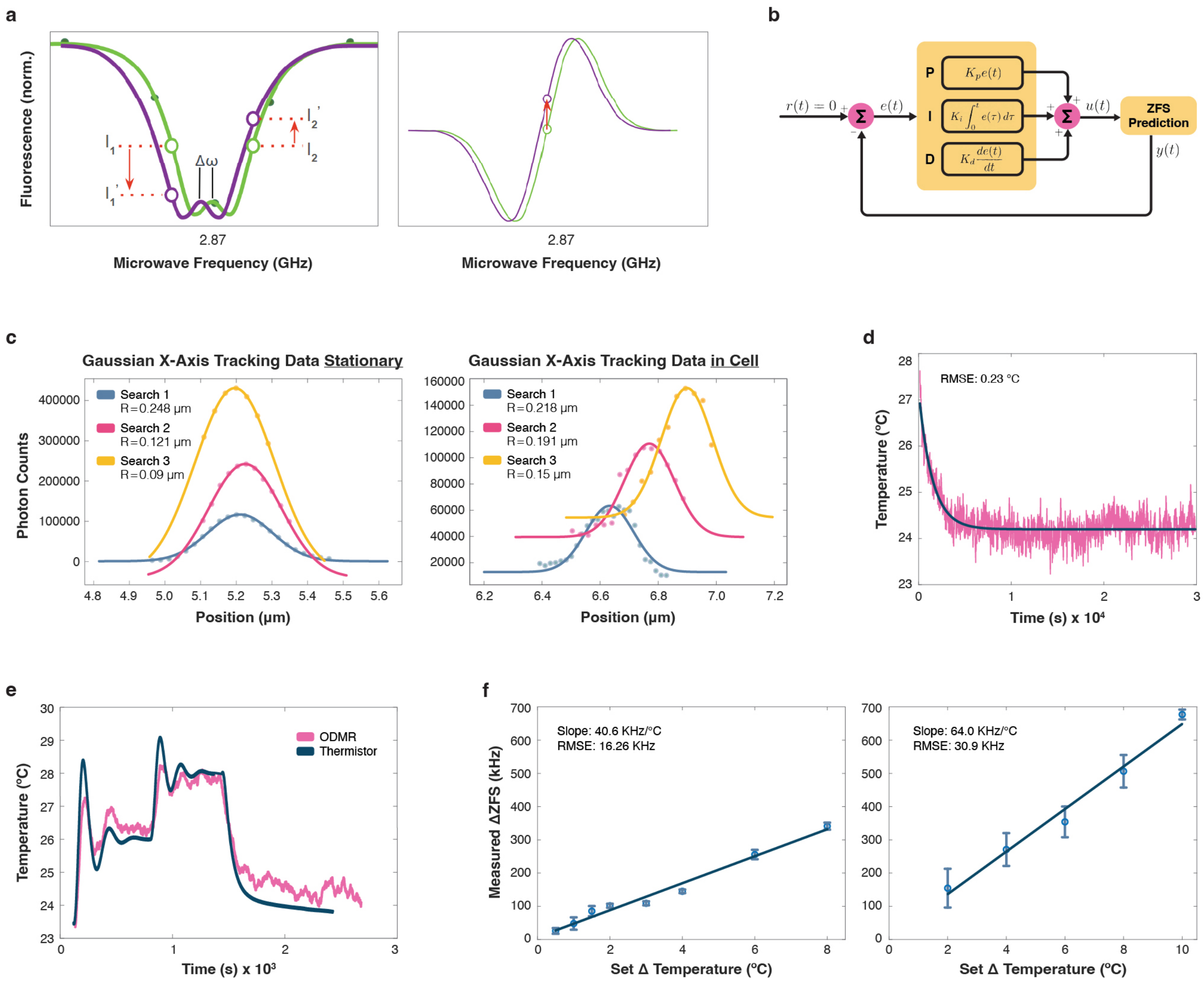

Figure E4: Methodology and performance of 2-point rapid ZFS tracking. (a) Illustration of the changes in the two probed frequencies, I_1 and I_2, upon a left shift in the ODMR spectrum. The left panel illustrates an initial (green curve) and a left-shifted (purple curve) spectrum. Subtraction of the initial spectrum from the shifted one (right panel) results in a quasi-linear regime in which the extracted quantity $I_2 - I_1$ increases and decreases upon a left or right shift, respectively. (b) A flow diagram demonstrating the PID logic in refining our ZFS prediction. (c) An example single-axis data from the implementation of our tracking algorithm measuring a stationary (left panel) and an intracellular (right panel) single diamond nanocrystal. The search radius changes to maximize signal, decreasing for slow moving particles and increasing for faster ones (see SI note S3). (d) ZFS tracking for a single diamond nanocrystal in air during a temperature drop. The blue line is the exponential fit $T(x) = c \cdot e^{-\frac{x}{t}} + T_a$, where T_a is the ambient temperature, c is the initial amplitude of the temperature deviation from T_a, and t is the characteristic time constant of the decay. A root-mean-square (RMS) error of 0.23 °C demonstrates the high precision of this approach at a high temporal resolution of 400 ms. (e) Simultaneous ZFS and thermistor data tracking during temperature modulation of a TEC controller, demonstrating the robustness of our PID rapid ZFS tracking. The differences in temperature between the ZFS and the thermistor data are mainly a result of a distance of a few millimeters between the thermistor and the nanocrystal. (f) Temperature dependence of a bare nanocrystal in air utilizing 50 s integration of our rapid ZFS tracking measurement using an oil (left panel) and an air (right panel) objective. Oil objective measurements were limited by a larger distance between the thermistor and the nanocrystal, leading to a lower set temperature dependence, but featured lower variation and higher precision ($RMSerror = 16.3$ KHz) compared with the air objective measurements ($RMSerror = 30.90$ kHz). Each point is comprised of 3 different measurements with error bars representing one standard deviation.

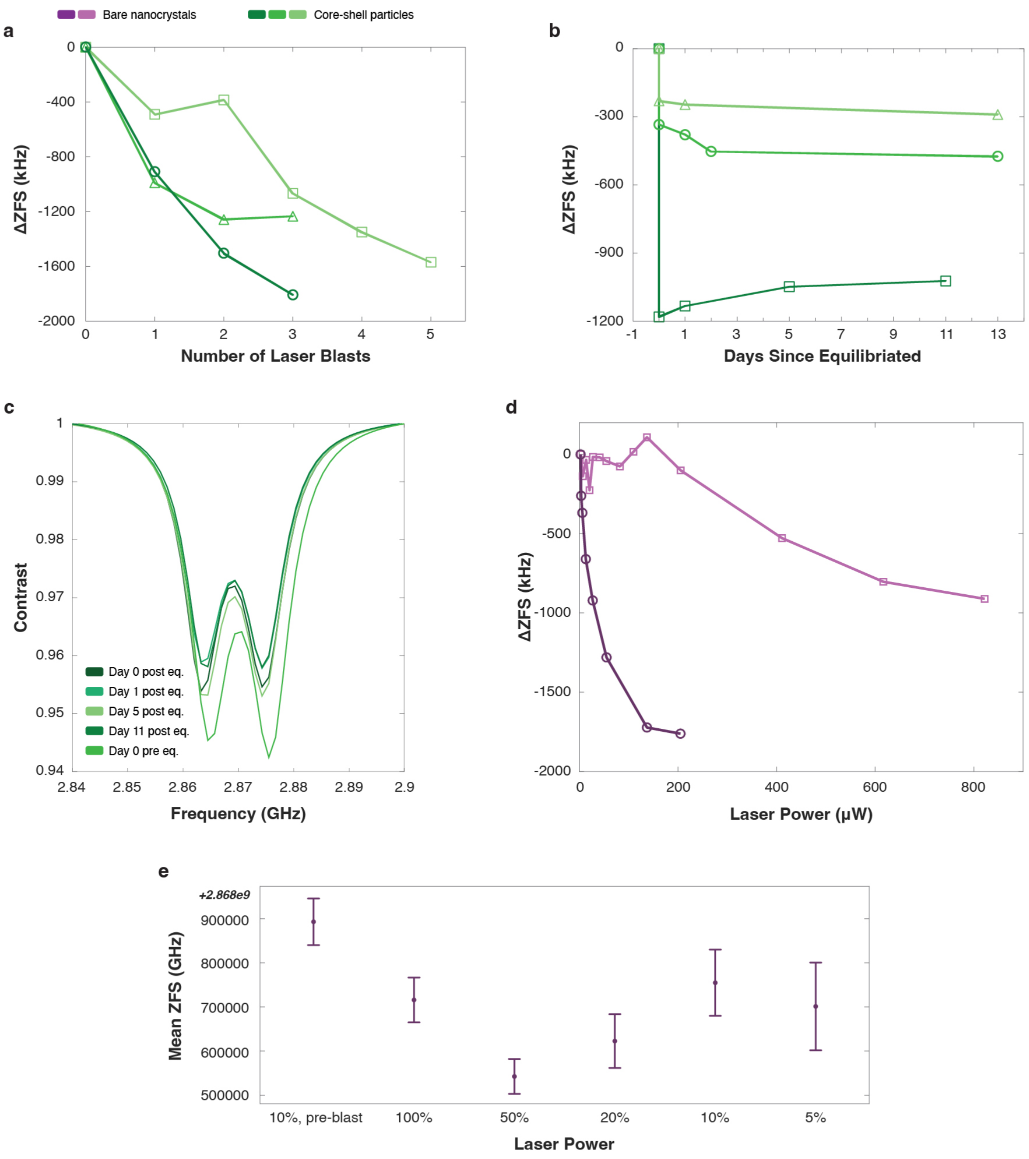

Figure E5: Further evidence for photo-induced charge transfer. (a) ZFS shifts in three different core-shell particles following consecutive 30 s laser illumination pulses at higher power (55 μW compared with 7-10 μW during measurements). Shifts on the order of 1 MHz are obtained within just a few pulses, compared with the thousands of seconds time scale shown in Extended Figure E3(b). Blasts at higher power achieved equilibration after a few seconds. (b) ZFS stability of three different core-shell particles up to 13 days following equilibration. The initial point at 0 KHz represents the initial ZFS before equilibration. The spectra in (c) show a representative core-shell nanoparticle before (Day 0 pre eq.) and days after (post eq.) equilibration. The spectra clearly demonstrate the shift towards lower frequencies and their stability over time, ruling out any temperature-related ZFS shifts. (d) Increasing ZFS shifts for two bare particles in PBS obtained from ODMR spectra taken at various laser powers. The end points represent the highest power at which we were able to obtain a reasonable contrast in order to fit the spectra. We were unable to achieve equilibration of ZFS for bare particles in PBS, implying that laser illumination over time, and not just the laser power, plays a role in the observed ZFS shifts. (e) The dependence of ZFS shifts in bare nanocrystals was measured as a function of % laser power (where 10%≈10 μW). The measurement showing no systematic shifts of ZFS serves as a control for the measurements in PBS (panel d) and as confirmation that the measurement is not perturbed by laser-induced heating.

Table E1: Summary of p-values from t-tests assessing significance between groups in toxicity and inflammation experiments and in the drift term analysis for ZFS time traces in PBS and in RAW cells.

**LDH assay**		10 µg/mL	50 µg/mL	200 µg/mL	
24 hrs	B40 vs C40	0.116	0.292	0.022	
	B70 vs C70	0.186	0.050	0.062	
6 hrs	B40 vs C40	0.619	0.373	0.241	
	B70 vs C70	0.337	0.169	0.151	
48 hrs	B40 vs C40	0.076	0.889	0.066	
	B70 vs C70	0.030	0.047	0.062	
**NF-κB assay**		10 µg/mL	50 µg/mL	100 µg/mL	200 µg/mL
Overnight	B40 vs C40	0.839	1.83×10^{-3}	0.010	
	B70 vs C70	0.205	0.305	0.099	0.085
	B40 vs Cells	3.59×10^{-8}	3.13×10^{-13}	7.49×10^{-20}	
	C40 vs Cells	7.49×10^{-9}	0.074	1.17×10^{-14}	
	B70 vs Cells	0.692	0.142	0.001	0.039
	C70 vs Cells	0.101	0.499	0.402	0.006
**TNF-α assay**		10 µg/mL	50 µg/mL	100 µg/mL	
Overnight	B40 vs C40	0.708	0.013	0.022	
	B70 vs C70	0.033	7.55×10^{-3}	1.56×10^{-4}	
	B40 vs Cells	0.019	6.23×10^{-4}	0.013	
	C40 vs Cells	0.139	0.013	9.13×10^{-5}	
	B70 vs Cells	0.068	0.003	1.81×10^{-6}	
	C70 vs Cells	0.074	0.021	0.023	
**Drift analysis**					
In PBS	Bare vs core-shell	0.018			
In RAW cells		Core-shell nascent	Core-shell LPS	Bare nascent	Bare LPS
	Core-shell nascent	X	0.15	0.84	5.74×10^{-6}
	Core-shell LPS	0.15	X	0.58	3.85×10^{-6}
	Bare nascent	0.84	0.58	X	6.38×10^{-3}
	Bare LPS	5.74×10^{-6}	X	6.38×10^{-3}	3.85×10^{-6}

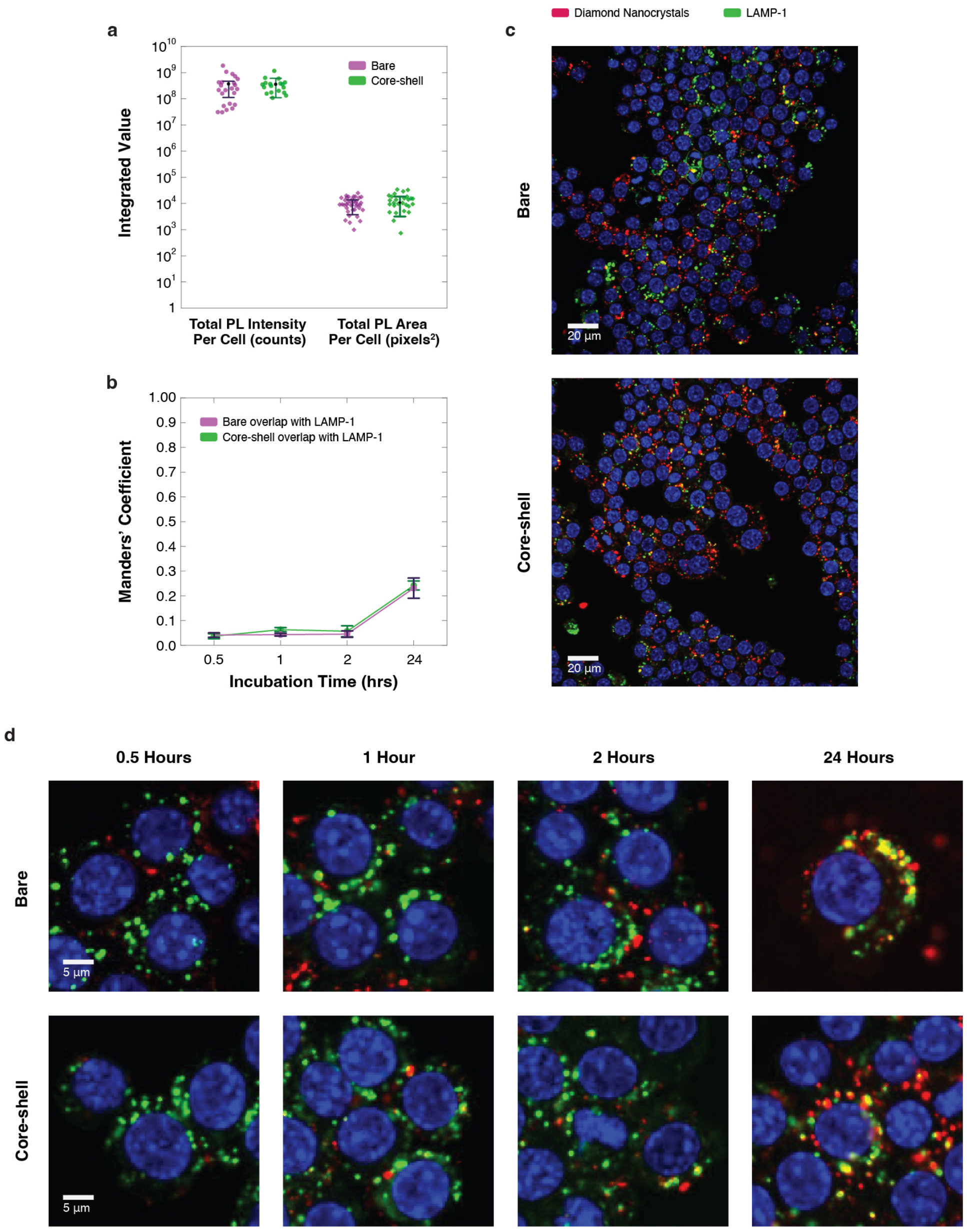

Figure E6: Cellular uptake and co-localization. (a) The fluorescence of bare and core-shell nanoparticles in terms of average area and intensity per measured cell, showing no significant difference ($p = 0.322$ and $p = 0.467$ for area and intensity, respectively). (b) Manders' coefficient representing the fraction of red fluorescence from bare and core-shell particles overlapping with green fluorescence signal from LAMP-1 labeled lysosomes after varying periods of incubation time. A similar ($p = 0.111$) increase to $\sim$25% overlap is seen in the co-localization of both particles at the 24 hrs time point. (c) Representative confocal figures from bare (top) and core-shell (bottom) showing internalized particles after 24 hrs incubation. (d) Representative zoomed confocal images of cells incubated bare (top) and core-shell (bottom) nanoparticles. Incubation period from left to right: 0.5, 1, 2, and 24 hours. Blue = DAPI; Green = Alexa Fluor 488 anti-LAMP-1; Red = diamond nanocrystals.

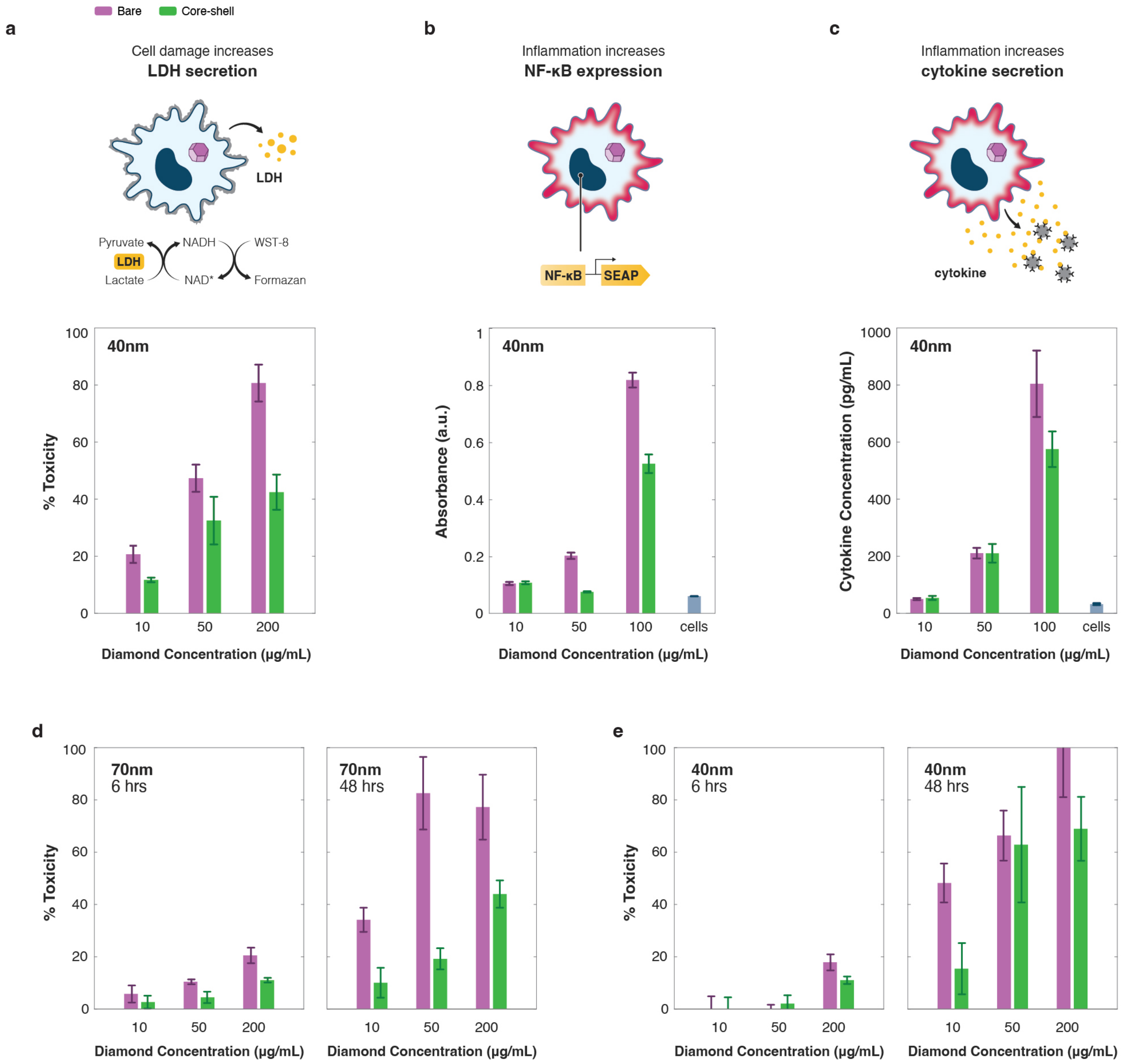

Figure E7: Additional toxicity and Inflammation data. (a-c) LDH (left panel), NF-κB (middle), and TNF-α (right panel) measurements from supernatant of RAW cells incubated with varying concentrations of 40 nm bare (purple) and core-shell (green) particles. Similar to our results with 70 nm particles, cells incubated with $\geq$50 µg/mL core-shell nanocrystals exhibited significantly lower (although not negligible compared to untreated cells) toxicity and inflammation compared to those incubated with similar bare nanocrystals. Top illustrations depict the key measured quantities in each assay. (d) 6 (left panel) and 48 (right panel) hours time points for the LDH toxicity assay for 70 nm particles. (e) 6 (left panel) and 48 (right panel) hours time points for the LDH toxicity assay for 40 nm particles. For a list of p-values, see Extended Data Table E1. We note that at the 6 hours time point, 70 nm particles exhibited higher toxicity compared with 40 nm for both bare and core-shell particles. Interestingly, after overnight incubation and longer time points, the toxicity was more dominant at the 40 nm particles. This might be due to the higher number of particles per unit mass, as well as the higher surface to volume ratio for 40 nm particles that might lead to aggregations.

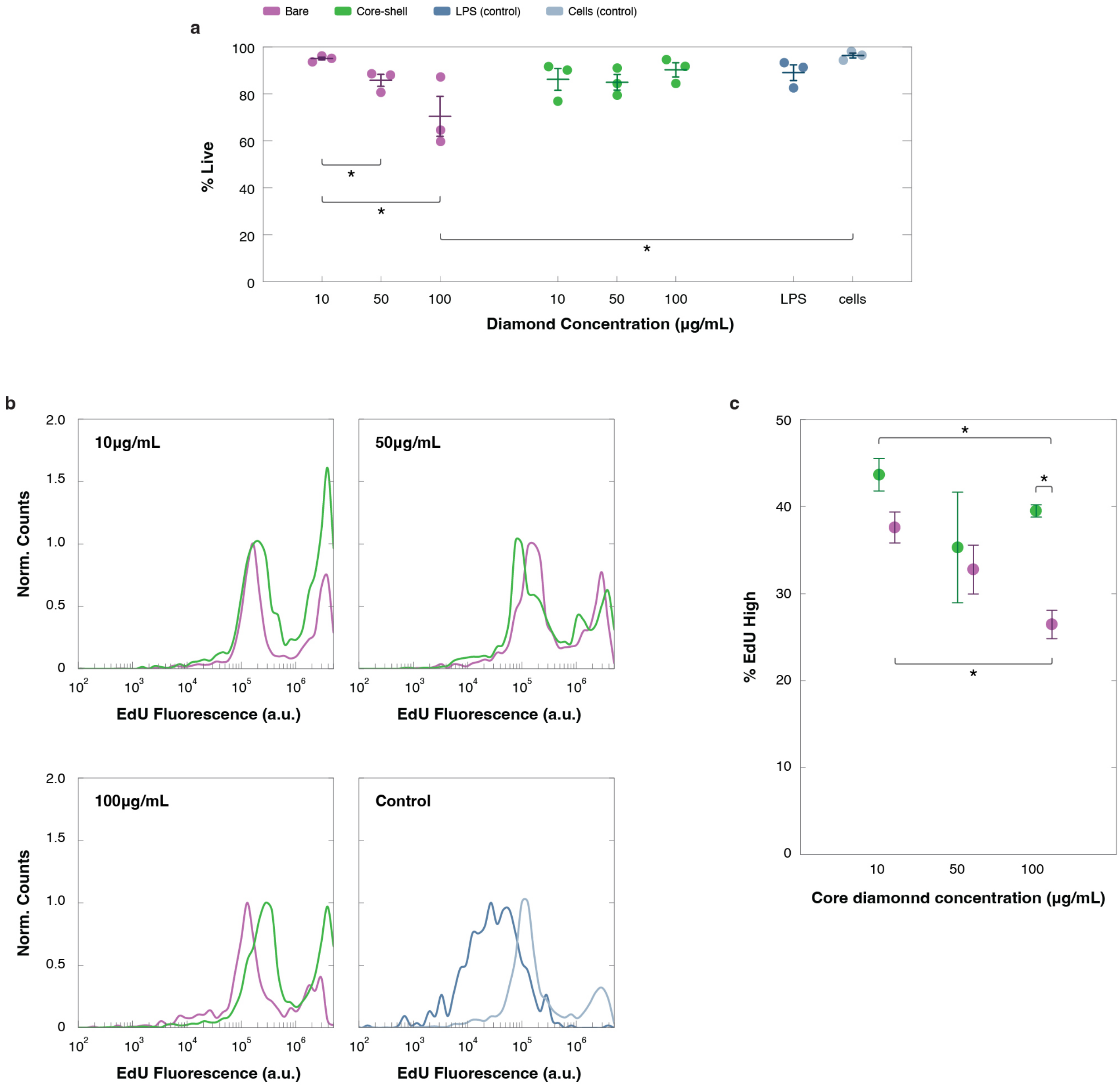
a
Bare
Core-shell
LPS (control)
Cells (control)
% Live
Diamond Concentration (μg/mL)
10
50
100
LPS
cells
b
10μg/mL
50μg/mL
100μg/mL
Control
Norm. Counts
EdU Fluorescence (a.u.)
c
% EdU High
Core diamonnd concentration (μg/mL)

Figure E8

Figure E8: Cellular viability and proliferation. (a) Cell viability assay obtained by flow cytometry after Zombie Aqua viability dye staining of cells incubated with bare and core-shell particles. The tested conditions showed no significant decrease in viability compared to control, excluding the 100 µg/mL bare particles group ($p = 0.039$). Both the 50 (p = 0.024) and the 100 µg/mL (p = 0.044) bare particles groups showed a slight yet significant decrease in viability compared to the 10 µg/mL bare group. (b) EdU incorporation studies for cells incubated with 10, 50, and 100 µg/mL core diamond of bare (purple curves) and core-shell (green curves) particles. The threshold for high EdU was determined at 500,000 and the fraction of high cells from the population was calculated (see Figure E9 for gating strategy). While cells incubated with both particle types showed robust DNA synthesis relative to LPS-treated cells, cells with core-shell particles showed increased levels compared to their bare counterparts. The histograms shown represent integration of all replicates. The fractions for cells showing high EdU expression after incubation with bare (purple) and core-shell (green) particles are shown in panel (c). Similar to the live-dead assay, most conditions showed no significant decrease in DNA synthesis. However, the 100 µg/mL bare particles group showed slight, yet significant decrease compared to the 100 µg/mL core-shell particles group (p = 0.017), as well as to both the bare (p = 0.020) and core-shell (p = 0.013) 10 µg/mL particle groups. The mean %EdU high values for 10, 50, and 100 µg/mL bare particle groups (n = 3) were 37.6±1.8%, 32.8±2.8%, and 26.5±1.6%, respectively. The mean % EdU high values for 10, 50, and 100 µg/mL core diamond in core-shell particle groups ($n = 2$) were 43.7±1.9%, 35.3±6.4%, and 39.5±0.7%, respectively.

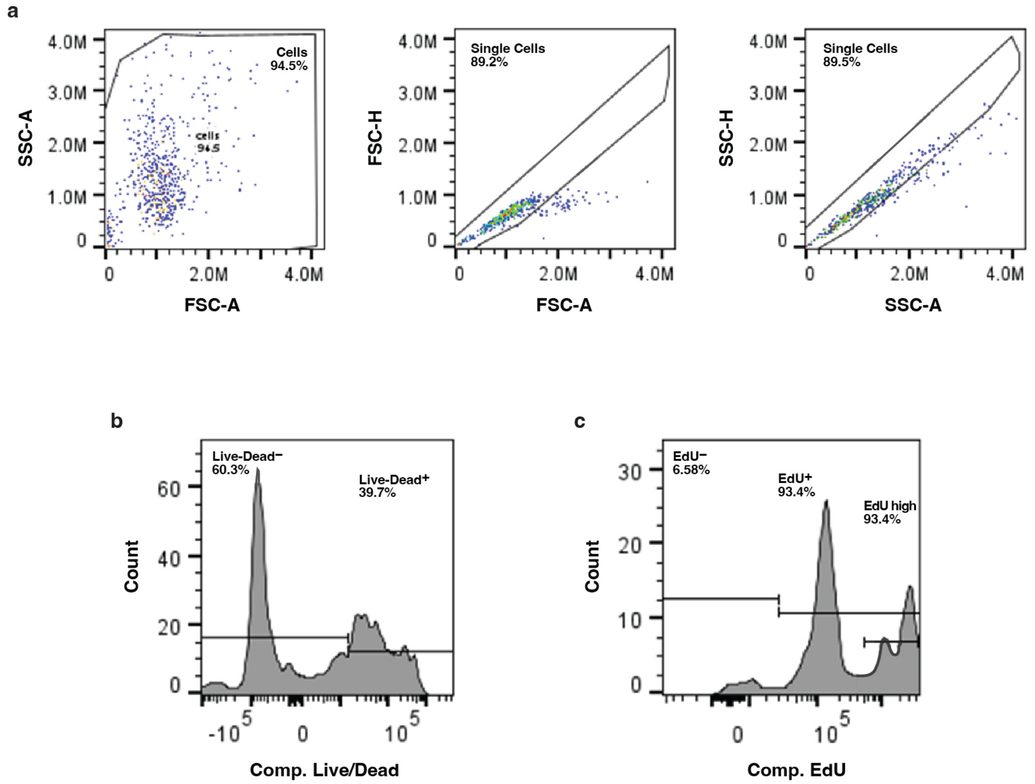

Figure E9: Flow cytometry gating strategy for viability and proliferation. (a) Cells were identified using forward and side scatter (left panel), then single cells were selected using forward scatter area and height (middle panel). Another round of single-cell selection was done using side scatter area and height (right panel). (b) A representative LIVE/DEAD histogram of cells incubated with 100 µg/mL bare diamond nanocrystals. Dead cells were excluded by selecting the LIVE/DEAD negative population. The percentage of LIVE/DEAD negative population was reported as live cells for the viability study in Figure E8. (c) A representative EdU histogram. The threshold for EdU High cells was determined at 500,000. Positive gates were set using fluorescence minus one (FMO) controls and unstained negative controls.

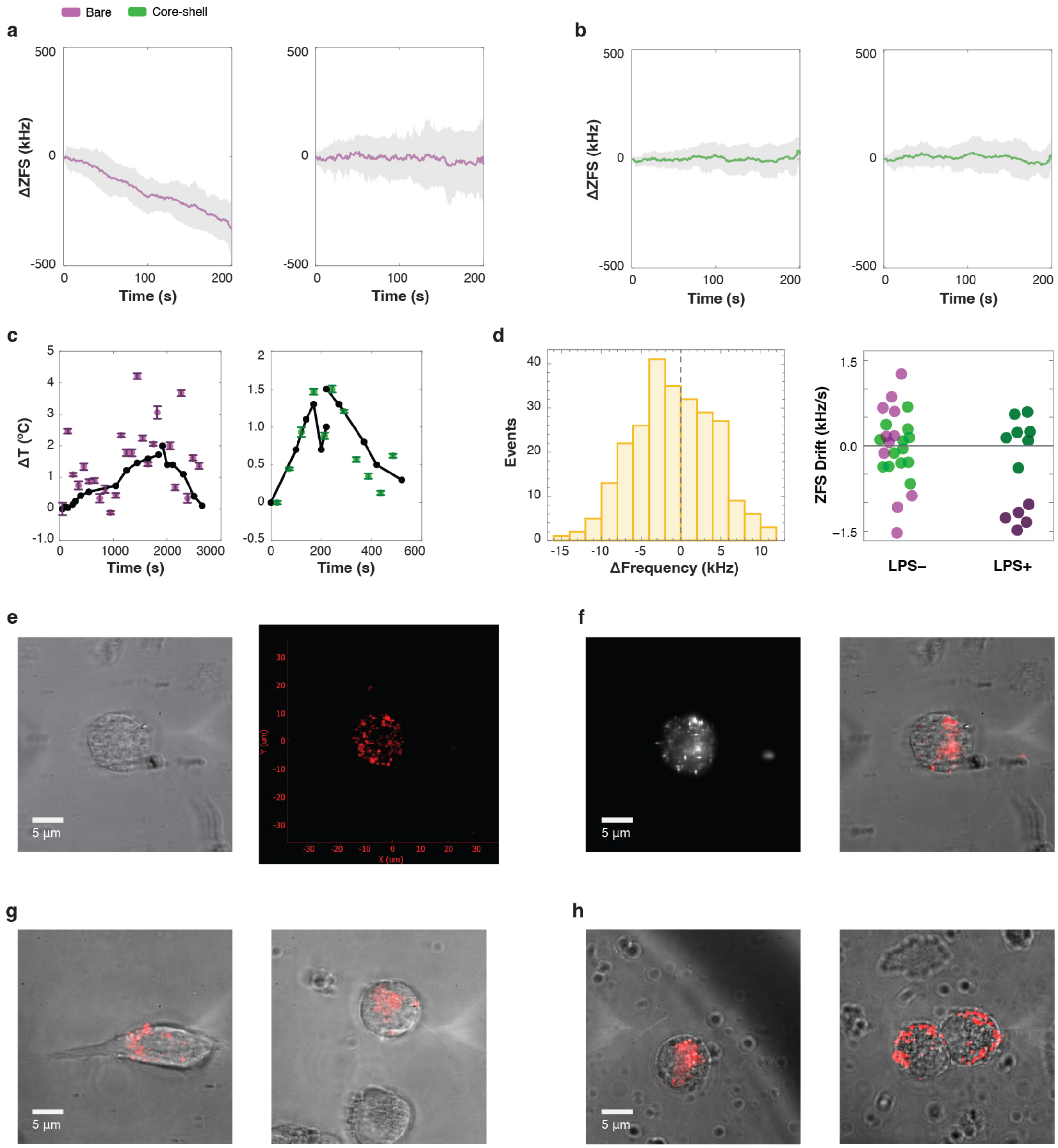

Figure E10: RAW cells ZFS measurements with bare and core-shell nanocrystals. (a) An average of all bare particles' ZFS time traces in inflamed (left panel) and nascent (right panel) RAW cells showing a clear distinction between the groups. (b) An average of all core-shell particles' ZFS time traces in inflamed (left panel) and nascent (right panel) RAW cells showing no qualitative change between the groups. In both (a) and (b), the gray shaded area represents one standard deviation. (c) ZFS tracking from bare (purple, left panel) and core-shell (green, right panel) particles inside a RAW cell. The points represent a 100 s integration during active modulation and monitoring (black lines) of the temperature of the solution (see methods). As expected, the core-shell particle exhibited an improved ZFS stability and followed the modulated temperature well (RMS error of 0.22 °C) compared to the bare particle (RMS error of 1.25 °C from the thermistor's measured temperature). See SI note S7 for details about this measurement. (d) Drift term analysis showing a representative error distribution histogram (left panel) for a bare particle ZFS time series taken in LPS-stimulated RAW cells. The deviation of the mean from zero represents a drift term. The right panel shows all the drift terms for intracellular bare (purple) and core-shell (green) particles. See Extended Data Table E1 for t-test results. (e) Bright field (left) and confocal (right) imaging of bare particles in a non-stimulated RAW cell. After background removal and applying thresholds, the confocal image is overlaid on the bright field image to produce Figure 4(a). An overlap (right panel) of the bright and fluorescence (left) wide-field images is shown in (f) for comparison. (g) and (h) show two representative overlap images for intracellular bare and core-shell particles, respectively.

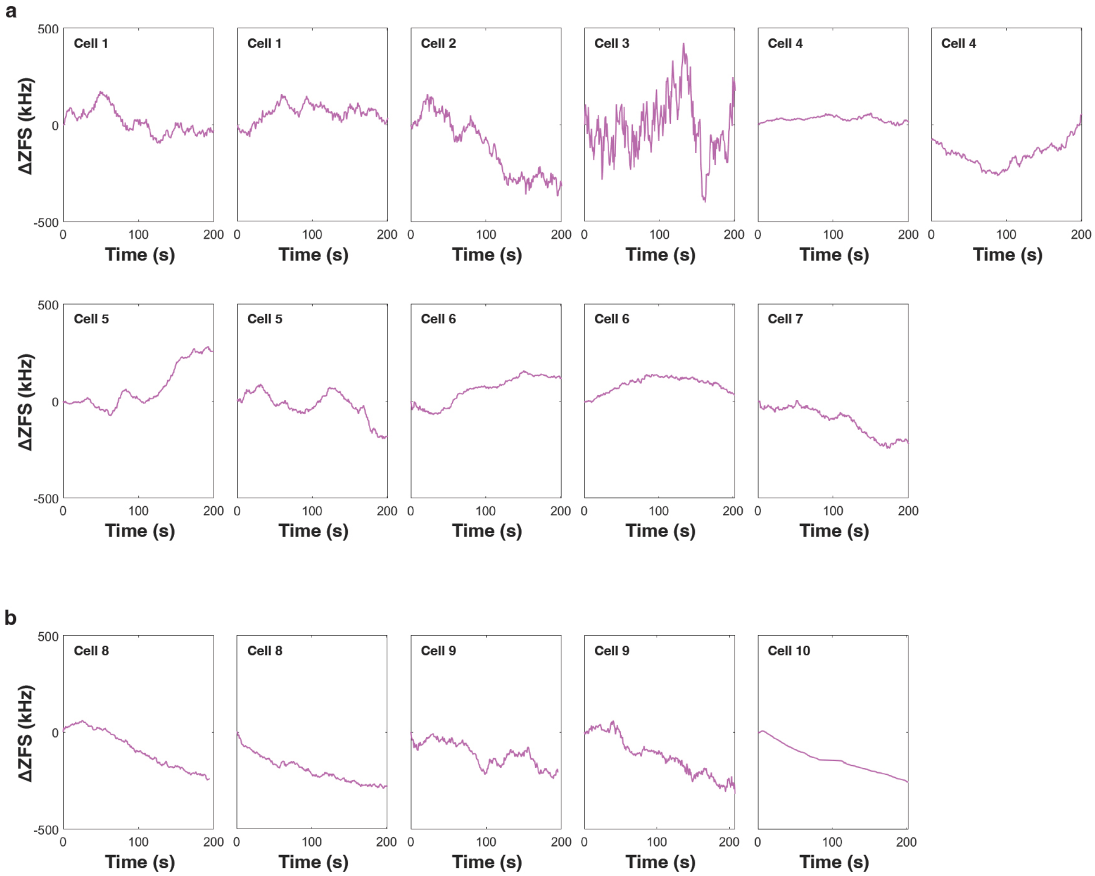

Figure E11: Individual data for ZFS tracking in lives cells with bare diamond nanocrystals. (a) 11 individual ZFS time traces from bare particles in resting (LPS-) RAW cells used for the left panel of Figure 4(d). (b) 5 individual ZFS time traces from bare particles in LPS+ RAW cells used for the left panel of Figure 4(f). Each data set is indicated with the cell number in which the measurement was taken. For example, the first 2 panels in (a) were taken from 2 different particles in cell 1. The large variability between particles in (a), is apparent across cells and within the same cell. See SI note S9 for further discussion.

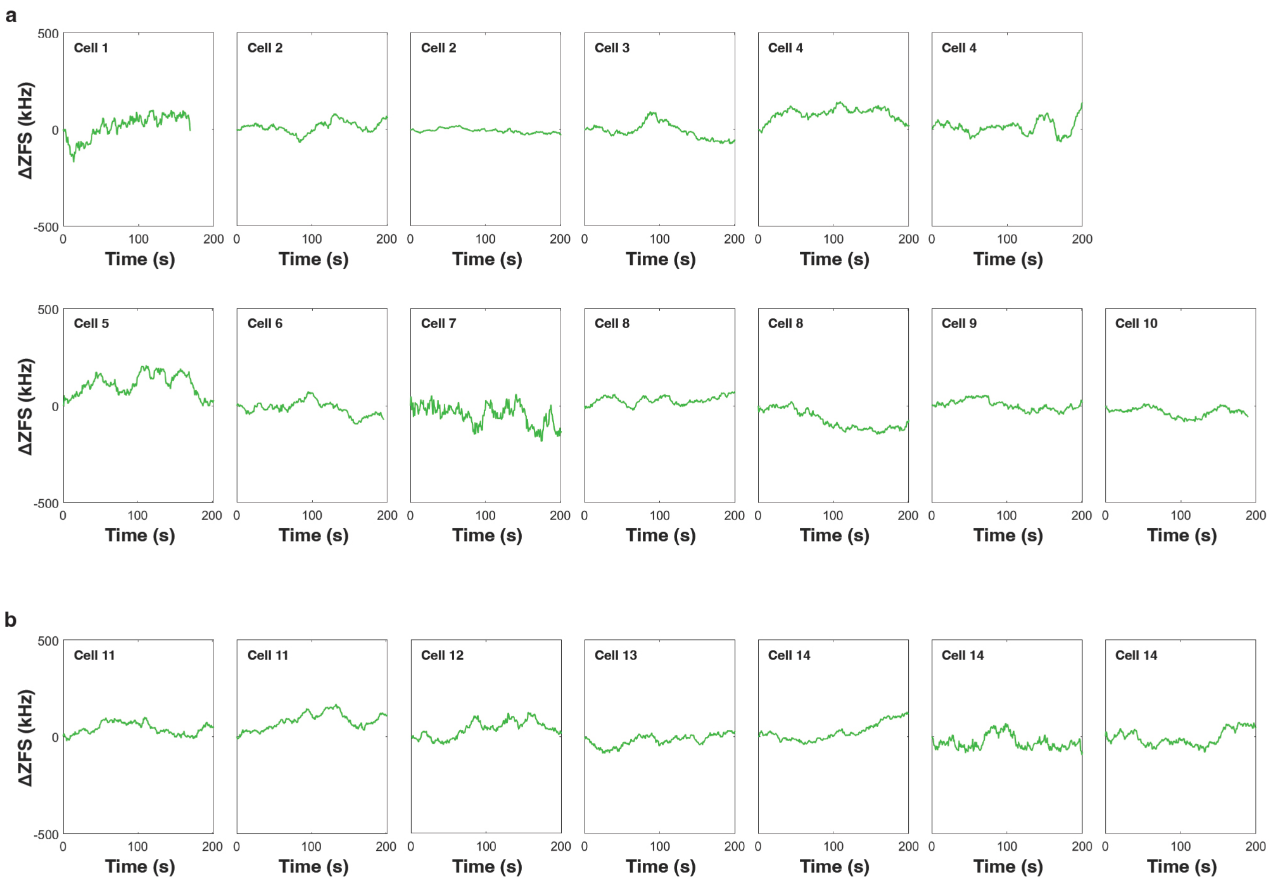

Figure E12: Individual data for ZFS tracking in lives cells with core-shell particles. (a) 13 individual ZFS time traces from core-shell particles in resting (LPS-) RAW cells used for the right panel of Figure 4(d). (b) 7 individual ZFS time traces from bare particles in LPS+ RAW cells used for the right panel of Figure 4(f). Each data set is indicated with the cell number in which the measurement was taken. For example, the first 2 panels in (b) were taken from 2 different particles in cell 11.

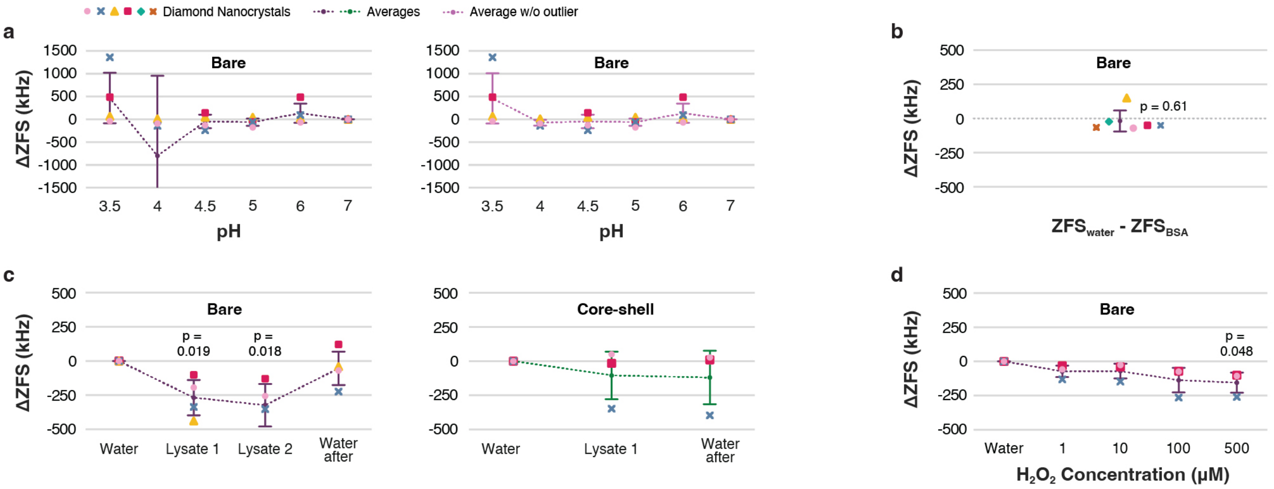

Figure E13: Investigating the biological mechanism for ZFS shifts in LPS-activated RAW cells. (a) Measurements of bare diamond nanocrystals ($n = 4$) in varying pH plotted as a change in ZFS compared to the measurement at pH 7. No significant changes were found across all pH conditions ($p = 0.391, 0.302, 0.642, 0.385$, and 0.281 for pH 6, 5, 4.5, 4, and 3.5, respectively). The measurement of nanocrystal 3 (red squares) at pH 4 was designated as an outlier by Tukey's method. Nevertheless, it was included in the reported significance values, as well as the average in the left plot (purple dotted line). For convenience, we added the average calculated without this outlier (right plot) as the light pink dotted line labeled "Average w/o outliers." (b) Measurements of bare diamond nanocrystals ($n = 6$) in 300 g/L BSA compared to initial measurements in water. While most ZFS values shifted towards lower frequencies, the shifts were small compared to those measured in inflamed cells and were not statistically significant ($p = 0.613$) compared to measurements in water, which were taken as the reference ZFS. (c) Measurements of bare ($n = 4$, left panel) and core-shell ($n = 3$, right panel) particles incubated with lysate of LPS-stimulated RAW cells. Two consecutive measurements for bare particles produced a significant ZFS shift compared to water ($p = 0.019$ and 0.018 for the 1st and 2nd measurements, respectively). The ZFS mostly recovered when washed and measured in water again ($p = 0.250$ compared to the initial water measurement). In contrast, measurements in lysate produced no significant change for core-shell particles ($p = 0.243$). (d) Measurements of bare ($n = 3$) particles incubated with varying concentrations of H_2O_2. ZFS shifted towards lower frequencies with increasing H_2O_2 concentration, as expected. Most measurements were only approaching significance ($p = 0.066, 0.101$, and 0.082 for 1, 10, and 100 μM, likely due to the small number of measurements. Nevertheless, the highest concentration showed a significant ($p = 0.048$) ZFS shift to lower frequencies.

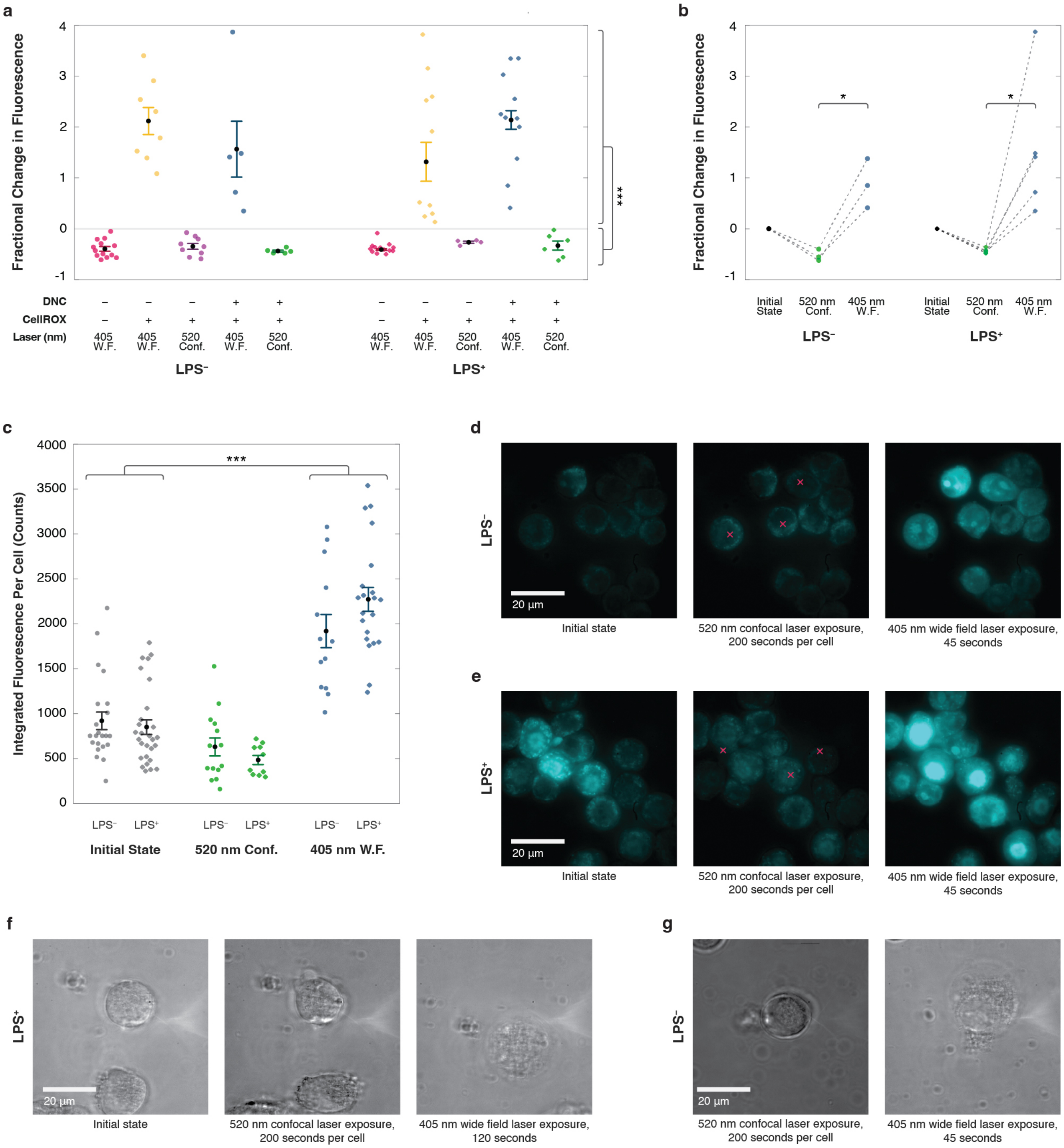
a
Fractional Change in Fluorescence
***
DNC
CellROX
Laser (nm)
405 W.F.
520 Conf.
LPS⁻
LPS⁺
b
*
Initial State
520 nm Conf.
405 nm W.F.
c
Integrated Fluorescence Per Cell (Counts)
Initial State
520 nm Conf.
405 nm W.F.
d
e
f
g
20 µm
Initial state
520 nm confocal laser exposure, 200 seconds per cell
405 nm wide field laser exposure, 45 seconds
405 nm wide field laser exposure, 120 seconds

Figure E14

Figure E14: Phototoxicity and ROS control assay. (a) Fractional change in integrated per cell fluorescence under different illumination regimes. We compared initial signal from LPS- (circles) and LPS+ (diamonds) cells to the signal following confocal illumination identical to our measurement conditions (200 s of 520 nm laser illumination at 8×10^3 W/cm^2 focused to a 400 nm spot). Cells were also imaged following incubation with diamond nanocrystals (DNC) to eliminate any influence of particle internalization. Positive controls were obtained by a 45 s illumination with a 405 nm laser at 1.5×10^3 W/cm^2 to produce a robust ROS signal. I all cases, a mild decrease in signal was observed after confocal illumination due to bleaching of autofluorescence signal (this effect was observed in unstained cells as well). A significant increase ($p < 0.001$) in signal was observed in all stained groups following 405 nm illumination, regardless of whether or not the cells contained DNCs. (b) ROS signal under sequential illumination of LPS- and LPS+ cells with 520 nm confocal (green), matching ZFS measurements conditions, followed by a 405 nm widefield illumination (blue) showing identical trend to panel (a). The robust and significant signal following 405 nm illumination for both LPS- and LPS+ cells ($p = 0.03$ compared to confocal illumination in both groups), confirms that confocal illumination does not interfere with ROS detection. (c) Raw, background-subtracted per cell intensity values for LPS- and LPS+ cells under different illumination conditions. The results show that compared to non-irradiated cells, there is no significant difference in ROS signal from cells irradiated with confocal 520 nm laser in our measurement conditions. However, a significant increase in signal is detected from cells undergoing 405 nm widefield illumination for both LPS- ($p = 2.00 \times 10^{-4}$ and $p = 1.22 \times 10^{-5}$ compared to initial and conf. illumination groups, respectively) and LPS+ ($p = 1.86 \times 10^{-10}$ and $p = 3.89 \times 10^{-12}$ compared to initial and conf. illumination groups, respectively) cells. We note that panel C integrates results from cells with and without DNCs. Interestingly, no robust ROS signal changes are detected between LPS- and LPS+ cells, probably due to the chosen time point (see SI note S10 for further discussion). (d) A representative example of ROS signal from non-irradiated LPS- cells (left panel), followed by 520 nm laser confocal irradiation of 3 distinct cells (red axes in middle panel), and then by a 405 nm laser widefield (W.F.) irradiation (right panel), matching the data presented in panel (b). (e) A representative example of ROS signal from non-irradiated LPS+ cells following the same sequential irradiation treatments. In both LPS- and LPS+ cells, a robust ROS signal appears after 405 nm W.F. irradiation, but not after 520 nm confocal irradiation. (f) A representative example brightfield image from non-irradiated (left panel) LPS+ cells following confocal irradiation (middle panel showing no apparent morphology changes in the irradiated cell) and a destructive, 405 nm W.F exposure for 120 s (right panel, showing clear morphological changes). (g) A representative example brightfield image from LPS- cells following confocal irradiation (left panel) and a destructive, 520 nm confocal exposure for 200 s at a power of 8×10^6 W/cm^2, 3 orders of magnitude higher than that used in our measurements (right panel, showing clear morphological changes). Morphology changes were not analyzed quantitatively and the images brought here are just for visual reference.

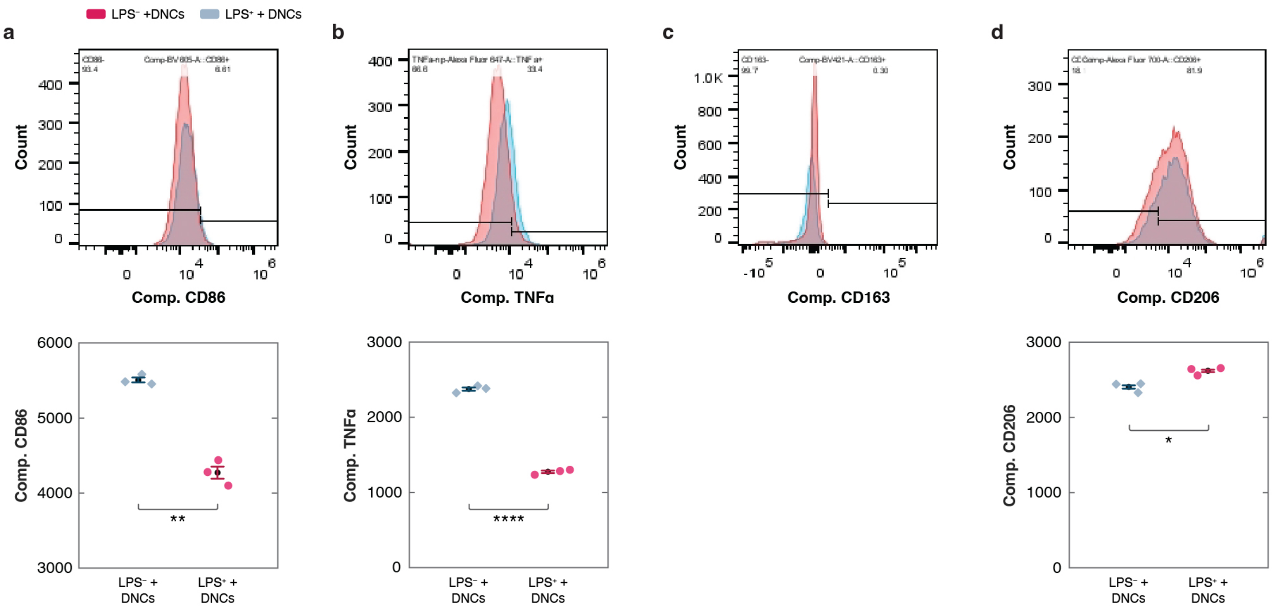

Figure E15: Macrophage polarization assay for LPS+ RAW cells. Histogram of CD86 labeling in the upper panel in (a) and the mean Fluorescence intensity (MFI) values (lower panel) show a significant increase in CD86 expression ($p = 0.003$) upon LPS stimulation of nanocrystals containing cells. (b) Histogram (upper panels) and MFI values (lower panels) of TNFα labeling showing a significant increase ($p = 1.183 \times 10^{-5}$) in TNFα expression upon LPS stimulation. Increase of both CD86 and TNFα is typical of LPS stimulation and polarization of cells towards M1 macrophages. (c) Histogram of CD163 labeling showing no detectable signal after background subtraction (MFI is negative). (d) Histogram (upper panels) and MFI values (lower panels) of CD206 showing no M2 characteristics in LPS-stimulated RAW cells containing nanocrystals. Interestingly, the tight distribution of the samples resulted in a mild, yet statistically significant decrease of CD206 upon LPS stimulation ($p = 0.014$). These results show a clear LPS stimulation profile with characteristics of population shift towards M1, but not M2. See Extended Figure E16 for polarization assay gating information. All graphs show the mean (black circle) and the standard error, which might be hidden when it is smaller than the data point.

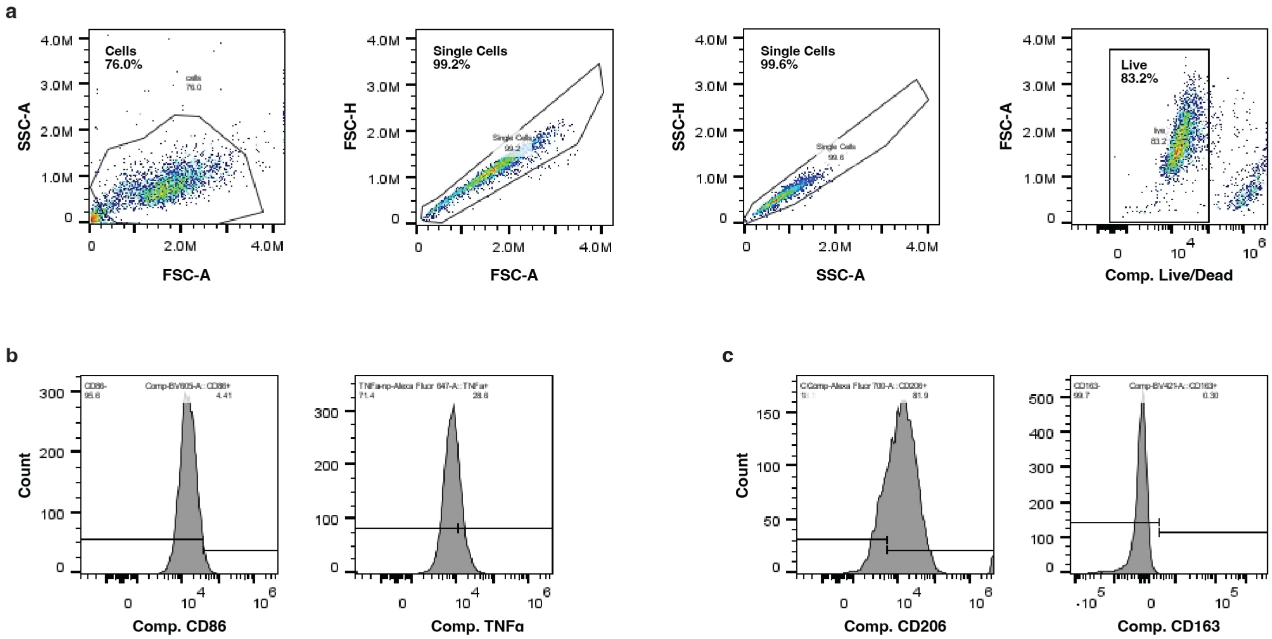

Figure E16: Flow cytometry gating strategy for macrophage polarization assay. (a) From left to right: cells were identified using forward and side scatter (1st panel), then single cells were selected using forward scatter area and height (2nd panel). Another round of single-cell selection was done using side scatter area and height (3rd panel). Dead cells were excluded by selecting the LIVE/DEAD negative population (4th panel). (b) Representative histograms of compensated readout of CD86 (left panel) and TNFa (right panel) as markers for typical LPS-induced inflammation with population shift towards M1 polarization. (c) Representative histograms of compensated readout of CD206 (left panel) and CD163 (right panel) as markers macrophages' response with population shift towards M2 polarization. Positive gates were set using fluorescence minus one (FMO) control and the unstained negative controls. However, MFI was chosen as the reported quantity for the histograms in Extended Figure E15.

Supplementary Notes

S1 Theoretical model

We aimed to develop a comprehensive model describing the interaction of NDs with their environment under conditions relevant to NV biosensing experiments. As such, our system is comprised of type 1b, oxygen-terminated NDs hosting an ensemble of NVs that are placed under laser excitation (Figure 1(a)). Conventional NV-based electric field sensing relies on the dipole moments $d_{\|}$ and $d_{\perp}$, which couple to the axial and transverse electric field components, respectively [1] (Figure 1(b)). In randomly oriented NV ensembles, these interactions are dependent on the relative orientation of each NV center. This orientation dependence leads to an averaging effect, resulting in ODMR line broadening [2] that varies randomly among different diamond nanoparticles, thus limiting their utility in sensing applications.

To address these limitations, we consider the full ground-state Hamiltonian in the $\{|+1\rangle, |0\rangle, |-1\rangle\}$ triplet basis [3,4]:

$$\frac{\mathcal{H}}{h} = \begin{pmatrix} \frac{D}{3} + \frac{d_{\|}}{3}E_z + \gamma B_z & \frac{d'}{\sqrt{2}}E_- + \frac{\gamma}{\sqrt{2}}B_- & -d_{\perp}E_+ \\ \frac{d'}{\sqrt{2}}E_+ + \frac{\gamma}{\sqrt{2}}B_+ & -\frac{2D}{3} - \frac{2d_{\|}}{3}E_z & -\frac{d'}{\sqrt{2}}E_- + \frac{\gamma}{\sqrt{2}}B_- \\ -d_{\perp}E_- & -\frac{d'}{\sqrt{2}}E_+ + \frac{\gamma}{\sqrt{2}}B_+ & \frac{D}{3} + \frac{d_{\|}}{3}E_z - \gamma B_z \end{pmatrix}, \tag{1}$$

where h is Planck's constant, γ the gyromagnetic ratio, $\mathbf{S} = (S_x, S_y, S_z)$ the spin-1 matrices, $\mathbf{B} = (B_x, B_y, B_z)$ the magnetic field, $\mathbf{E} = (E_x, E_y, E_z)$ the electric field, $E_{\pm} = E_x \pm iE_y$, $B_{\pm} = B_x \pm iB_y$, and D the temperature-sensitive zero field splitting (ZFS). Importantly, we include the commonly neglected d' dipole term, which couples the $|0\rangle$ state to $|\pm1\rangle$ states (Figure 1(b)) and is estimated to be of similar magnitude as $d_{\perp}$ [2,5,6]. Using perturbation theory, we calculate the leading order (second-order) influence of d' on the transition frequencies of our ODMR spectra

$$f_{|0\rangle\to|\pm\rangle} = D + d_{\|}E_z \pm |d_{\perp}E_{\perp}| + \frac{|d'E_{\perp}|^2}{2D}[3 \pm \cos(3\phi)], \tag{2}$$

with $E_{\perp} = \sqrt{E_x^2 + E_y^2}$ and $\phi = \arctan(E_y/E_x)$. Considering ZFS $= (f_{|0\rangle\to|+\rangle} + f_{|0\rangle\to|-\rangle})/2 - D$, and the ensemble-average ($\langle ... \rangle$) yielding $\langle E_z \rangle \to 0$, the effects of the first-order E-field terms cancel, limiting their implementations in electric field sensing. In contrast, the dependence of the ZFS on d' yields $\langle \text{ZFS} \rangle = 3\left\langle |E_{\perp}|^2 \right\rangle |d'|^2/2D$. It is clear, therefore, that d' unlocks a new possibility of sensing electric fields with an ensemble of NVs via shifts of the ZFS.

To account for the electric field experienced by each NV, we follow the procedure described in ref. [7] and solve the Poisson's equation, accounting for the implanted nitrogen/P1, vacancies, and NVs [8,9] (Extended Figure E1). Figure 1(c) describes the P1 densities (upper panel) and the resulting electric field profiles (lower panel) for a spherical particle with surface potentials $\phi_S = 0.5$ V, corresponding to the expected potential on a bare, oxygen-terminated nanocrystals [7], and $\phi_S = -0.5$ V to model an environment-induced shift.

Finally, we apply a Lindblad formalism to obtain an ODMR simulation for 100, randomly oriented NVs under laser excitation and Rabi driving. Figure 1(d) illustrates the corresponding spectra for $\phi_S = 0$ V and $\phi_S = 0.5$ V with and without accounting for the influence of d'. The clear shift in the spectra confirms our analytical results in Eq. 2. To demonstrate the effect on ZFS measurements, we use our Lindblad formalism to plot the ZFS dependence on the electric field magnitude at the surface for various possible values of d' (Figure 1(e)).

S2 ODMR fitting and extraction of dipole terms

ODMR fitting is done using a double Lorentzian of the form:

$$f(x) = \frac{A_1}{1 + \left(\frac{2(x-x_1)}{\gamma_1}\right)^2} + \frac{A_2}{1 + \left(\frac{2(x-x_2)}{\gamma_2}\right)^2} + y_0, \tag{3}$$

where the fitting parameters x_1 and x_2 represent the f^- and f^+ transitions, A_1 and A_2 represent their respective contrasts, γ_1 and γ_2 represent their respective FWHM, and y_0 represents the total PL baseline without MW modulation. This form fits well for a single NV, yet our system is comprised of a 100 NVs, each has one of eight

orientations and a random position within the nanocrystal. As such, each experiences a different electric field vector and corresponding influences of the dipole moments. As captured well in our model (Figure 1 and SI note S1), the orientation-dependent interactions with the MW AC magnetic field and band bending induced electric field give rise to different broadening, contrast, and eigenstates for each of the NVs. This results in an asymmetric PL spectrum that diverges from the double Lorentzian fit in equation 3. While interactions that are independent of the field's sign (i.e., the d' and $d_\perp$ dipole moments) should not average out, the complex PL spectrum limits our ability to extract the compounded f^- and f^+ transitions and determine the appropriate dipole terms.

Interestingly, when fitting experimental ODMR data, the obtained ZFS left shifts were consistent, while the f^- to f^+ splits determined by $d_\perp$ presented high variability and no consistent trend (Extended Figure E2 and E3). This is possibly due to the overall broadening caused by $d_\perp$ [2], which isn't easily distinguished from other noise sources like AC magnetic fields. We emphasize here that while our experiments where inspired by the model, the limitations in the extraction of the Lorentzian features prevents us from making any quantitative determinations of the electric field changes during our experiments. We hope that collecting additional data will allow us to refine our model (for example: including the probability to lose the NV charge state during laser excitation, and considering changes in spin T_1 and optical lifetime in various NVs during our measurement).

S3 Rapid ZFS tracking

2-points measurement

We employ a 2-point measurement scheme [10] to estimate the zero-field splitting (ZFS) frequency, ω_{ZFS} of our NV center over time. In this scheme, given a center frequency ω_c, we measure the fluorescence at two microwave frequencies, $\omega_1 = \omega_c - \frac{\gamma}{2}$ and $\omega_2 = \omega_c + \frac{\gamma}{2}$, where γ is the full-width half maximum of the optically detected magnetic resonance (ODMR) spectrum of the NV. The fluorescence values at these frequencies are labeled I_1 and I_2. The choices of ω_1 and ω_2 are in the quasi-linear regime of the ODMR Lorentzian spectrum, so that changes in the values of I_1 and I_2 correspond linearly with changes in ω_{ZFS}. To utilize this property, we plot the "frequency jump" function, $f(\omega_c) = I_2(\omega_c) - I_1(\omega_c)$ (this function evaluates to 0 at $\omega_c = \omega_{\text{ZFS}}$ due to symmetry to the Lorentzian). The slope of this function near its zero is quasi-linear due to the aforementioned properties of the Lorentzian, and so for any given deviation in $I_2 - I_1$ reading from 0, we can compute the change in ZFS, ω_{ZFS}, as

$$\Delta\omega_{\text{ZFS}} = -\frac{I_2 - I_1}{\alpha}, \tag{4}$$

where α is the quasilinear slope at the zero point.

Once the quasilinear slope has been characterized, we implement sideband modulation to enable rapid switching between ω_1 and ω_2, centered around the initial ω_{ZFS}. Rather than directly measuring $\Delta\omega_{\text{ZFS}}$ as outlined in Eq. 4, we input this value as a "process variable" into a PID control loop to mitigate noise and fluctuations in the readings. In this setup, the set point error for the ZFS reading is defined as $u(t) = 0$, the predicted $\Delta\omega_{\text{ZFS}}$ value (process variable) is represented by $y(t)$, and the error fed into the PID computation is $e(t) = -y(t)$. This feedback loop effectively stabilizes the predicted $\Delta\omega_{\text{ZFS}}$ to have a 0 error, reducing jitter and suppressing the effects of noisy readouts. Running this process continuously over our measurement, we generate estimated ω_{ZFS} values over time.

Spatial Tracking of the NV

Fluorescence measurements of the NV center are highly sensitive to the focus of the confocal microscope. In dynamic environments, such as those involving fluctuating temperatures and moving fluids (e.g., within cells), it is necessary to implement a tracking system capable of concurrently acquiring fluorescence data for ZFS prediction while monitoring the NV center's position over time to refocus the measurement stage. To do this, we perform a sweep along the three spatial axes, centered at the previous focus position, with a sweep radius R. For example, along the x-axis, the sweep spans the interval $[x_0 - R, x_0 + R]$, where x_0 is the previous x-axis focus position. At each point along the sweep, we measure the fluorescence counts $I_{1,2}$. The fluorescence profile of the NV center, modeled as a point light source, exhibits a Gaussian distribution. Therefore, the total fluorescence collected at each point is used to estimate the new center position of the NV along the corresponding axis by fitting the measured

fluorescence profile to a Gaussian function. Meanwhile, since the $I_{1,2}$ measurements are taken over equal durations at each position, we can sum them up individually and use them to compute ω_{ZFS}.

Furthermore, we implemented a PID control loop to lock the sweep radius R, maintaining it at twice the drift length of the NV center measured in the previous observation along each spatial axis. Extended Figure E4 features examples of x-axis sweeps showing an increase in PL as the sweep-radius decreases showing convergence on the position of a stationary NV. In contrast, when the NV center is moving within a cell, the fluorescence data reveals a shift in the x-coordinate of the NV across multiple sweeps. Although R decreases in this case as well, it does not reach as small a value as in the stationary scenario due to the motion of the NV. Sweeps are performed sequentially along all three axes, with the coordinates of the other two axes held constant while sweeping along one axis, ensuring precise localization along each dimension.

S4 Evidence for photo-assisted charge transfer

While the charge transfer between the diamond and the environment is linked to the band bending profile, the existence of non-resonant laser excitation may promote this effect, and even lead to the ionization of defect states that otherwise would remain occupied. The suppression of the electron transfer process in core-shell particles is likely a combination of the protection of the surface from the environment and the energy barrier that reduces the probability of electron tunneling. Such an effect should scale exponentially with the thickness of the shell and should be further explored. While we cannot conclusively determine that laser excitation is necessary for the charge transfer to happen in the conditions measured here, our findings suggest that the reported ZFS shifts are promoted by photo-excitation. Extended Figure E5 shows several supporting evidence, including the laser-power dependence of the ZFS shifts of bare particles in PBS (Extended Figure E5(d)), as well as the effects of high-power laser exposure on the initial equilibration of core-shell particles (Extended Figure E5(a-b)). Particularly interesting is the lower frequency shift experienced by core-shell particles during equilibration, which suggests that electrons might initially flow into the diamond during this process, as the positive electric field is decreasing. This is in contrast to bare particles, which likely experience an electron flow out of the diamond as their electric field turns less negative. This is further supported by the PL profiles in Extended Figure E2, showing the stability in PL in core-shell particles vs the decrease in bare particles with increased duration of illumination.

S5 Toxicity and Inflammation

LDH assay

Following incubation with bare or core-shell particles and transfer of 50 µL of supernatant to a new flat-bottomed 96-well plate, 50 µL of Reaction Mixture (kit Cat. No. C20300) was added to each sample well and mixed well. The mixture was shaken in the dark at RT for 30 minutes. Subsequently, 50 µL of Stop Solution was added to each well, followed by mixing. Absorbance was measured at 490 nm from which the absorbance at 680 nm was subtracted. For maximum and minimum LDH activity, we incubated cells with 10 µL 10X Lysis buffer or 10 µL sterile water, respectively, instead of incubation with NDs. We calculated $\%\text{Toxicity} = \frac{\text{Sample activity}-\text{spontaneous activity}}{\text{Max activity}-\text{spontaneous activity}}$.

NF-κB assay

Following incubation with bare or core-shell particles and transfer of 20 µL of supernatant to a new flat-bottomed 96 well plate, 180 µL of QUANTI-Blue™ solution was added to each sample well and mixed well. The mixture was incubated at 37°C for 3 hours, and absorbance was measured at 620 nm from which the absorbance at 680 nm was subtracted. As a control, absorbance was measured from cells with no diamond nanocrystals.

Inflammatory cytokine secretion

Murine RAW Blue™ macrophages were incubated with bare or core-shell particles for 12 hours (overnight). The resulting supernatant was analyzed for cytokine secretion using a LEGENDplex™ Mouse Inflammation Panel (13-plex) kit (BioLegend, Cat. No. 740150) according to the manufacturer's instructions. In brief, standard curves for cytokine concentrations were created using the manufacturer's Standard Cocktail, which was diluted as instructed.

For each experimental well, 15 µL of supernatant were added to the provided 96-well V-bottom plate and mixed with 10 µL of Assay Buffer. Subsequently, 25 µL of Capture Beads and additional Assay Buffer were added to both standard and experimental wells. The plate was sealed, protected from light, and incubated with shaking at 700 rpm for two hours. Following the initial incubation period, the plate was centrifuged at 1050 rpm for five minutes. The resulting supernatant was removed, and the remaining beads were washed with 200 µL of 1X Wash Buffer. Centrifugation was repeated at 1050 rpm, and the Wash Buffer was removed. Next, 25 µL of Detection Antibody solution was added to each well. The plate was sealed, protected from light, and incubated with shaking at 700 rpm for one hour, after which 25 µL of SA-PE were added directly to each well without washing. The plate was sealed, covered, and incubated for an additional 30 minutes with shaking at 700 rpm. The plate was then centrifuged at 1050 rpm, and the beads were washed with 200 µL of 1X Wash Buffer. Centrifugation was repeated at 1050 rpm, and the Wash Buffer was carefully removed. Finally, the beads were reconstituted in 150 µL of Wash Buffer, and the plate was analyzed using a flow cytometer (Agilent ACEA NovoCyte® 1000).

S6 Thermodynamic analysis of RAW cell thermogenesis

To verify that the observed 268±29 kHz ZFS shift in bare nanodiamonds does not stem from an actual temperature increase, we employ thermodynamic considerations to demonstrate that such a variation is not physically attainable within a cellular environment. We follow the dimensional analysis approach for steady-state conditions in a dense medium, as presented in [11], which states that to the correct order of magnitude:

$$\Delta T \sim \frac{P}{\kappa L}$$

Here, ΔT represents the temperature increase at a given site, P is the input power from the heat source, L is the characteristic scale of the heat source, and κ is the thermal conductivity of the surrounding medium. Assuming an aqueous environment, $\kappa \sim 1\,\mathrm{W\,m^{-1}\,K^{-1}}$. The observed ZFS shift corresponds to a temperature change $\Delta T \sim 4\,\mathrm{K}$ over 200 s. Finally, to estimate the minimum power required for inducing a temperature change, we consider the most favorable scenario by selecting the smallest cellular structure capable of generating heat—the mitochondrion with $L \sim 1\,\mu\mathrm{m}$—as the heat source. This corresponds to a power of $P \sim 20\,\mathrm{nW}$ delivered in the cell by the heat source. However, the typical thermal power generated by a cell is approximately $\sim 100\,\mathrm{pJ}$ [12], making the value estimated here implausible, as it exceeds observed values by two orders of magnitude. As such, we exclude the possibility of the zero-field splitting (ZFS) shift arising from temperature-changing processes within the cell.

S7 ZFS tracking during temperature modulation

While no systematic shifts were detected in nascent cells, the substantial fluctuations present a major challenge to thermometry applications. We demonstrate the challenge of decoupling temperature measurements from the effects reported here by manipulating the temperature of the stage-top chamber using a high-precision resistive heater (Thorlabs Inc., model HT19R). We simultaneously tracked the coverslip temperature and the ZFS of a bare and a core-shell particle in nascent RAW cells (Extended Figure E10(c)). We determined the measured temperature by integrating 100 seconds of ZFS tracking data. Core-shell particles followed the temperature well with an average standard deviation of 0.25 °C, with an RMS error of 0.22 °C from the thermistor's measured temperature. In contrast, bare particles presented an average standard deviation of 1.12 °C and a significantly larger RMS of 1.25 °C from the thermistor's measured temperature.

S8 Coplanar waveguide

In experiments involving cells, microwaves were delivered using a lithographically fabricated coplanar waveguide. The waveguide is fabricated by defining a waveguide design on a 200 μm thick glass microscope coverslip. Following the lithographic patterning, 50 nm Ti, 1200 nm Cu, and 100 nm Au are deposited on the top before lifting off the underlying photoresist to create an Ω shaped antenna with a line width of 20 µm and a curvature radius of 200 µm. The waveguide is designed to be impedance matched (50 Ω) and to deliver microwaves to the opposite (clean glass with no metal) side of the coverslip. This is done to prevent metal contamination of the cells and to create a

large, uniform area of microwave field. To perform the experiments, the coplanar waveguides are mounted onto custom-made printed circuit boards (PCB) using a low-temperature indium-based solder.

S9 ZFS measurements in live cells

The data shown in Figure 4(d,f) in the main text features only four representative traces selected from a total of a larger set of n particles measured across N cells. Figures E11 and E12 for data from individual particles and assignment to the cell they were measured in. For example, measurement of two independent particles were made in four of the seven LPS- cells incubated with bare diamond nanocrystals.

Although measurements of multiple particles within a single cell open the possibility to investigate intracellular spatial (sub-cellular compartmentalization) dependence of the ZFS signal, such an investigation is beyond the scope of this study. Work that includes simultaneous investigation of ZFS signal from several internalized particles in currently in progress.

S10 Phototoxicity and ROS control assay

Cells were prepared in an 8 Well slide (µ-Slide 8 Well high, ibidi) with or without diamond nanocrystal according to the protocol detailed for ZFS measurements in live cells. Post incubation, half the wells were treated with 1.5 µg/mL LPS in complete media for 60 mins before adding 5 µM of ROS detector CellROX green and incubating for 30 min at 37 °C, according to the manufacturer's protocol [13]. Cells were washed 3 times before fluorescence images were taken on our home-built microscope. Images were processed via ImageJ. ROIs from background-subtracted images were extracted manually by tracing cell edges, and the total fluorescence signal from the pixels of each ROI was summed for non-irradiated cells (I_{before}), and cells undergoing 520 nm confocal or 405 nm widefield irradiation (I_{after}). As noted in the method section, the fractional change for each was computed as $(I_{after} - I_{before})/I_{before}$. We note that most cells were imaged under just one irradiation regime. Cells that undergo sequential irradiation with both 520 nm confocal and 405 nm widefield are presented in panel (b) of Figure E14. Unstained replicates were used as negative controls.

In all cases, confocal irradiation under the conditions of our measurements did not produce any increase in ROS signal and did not interfere with the robust and significant ROS signal produced following 45 s continuous 405 nm widefield irradiation. Interestingly, no robust ROS signal changes are detected between LPS- and LPS+ cells. We note that this can be a result of the chosen time point for imaging (60 min following LPS exposure) and is consistent with results from literature [14, 15]. It is possible that if ROS are involved in the real-time ZFS shifts reported in this manuscript for LPS+ cells, their influence on the sensor persists after the ROS levels in the cells have decreased. Alternatively, while some evidence suggests otherwise, ROS might not be the main factor producing the ZFS shifts reported here. An in-depth mechanistic study designed to answer these and other mechanistic questions is currently ongoing.

S11 Core-Shell synthesize

The growth of Silica shells on diamond nanocrystals was performed using a previously described process utilizing a sol-gel Stöber process [16] with a tetraethyl orthosilicate (TEOS) precursor. A 1 mg/mL of as-purchased carboxylated 70 nm diamond nanocrystals were sonicated for 20 min. Meanwhile, 8 mg PVP (10 kDa; Sigma-Alderich) was dissolved in 16.5 mL reversed-osmosis purified H_2O (MQ-H_2O) and sonicated for 10 min. The nanocrystals were added into the PVP solution and stirred at 600 rpm overnight. The synthesized PVP-nanocrystals were centrifuged at 20000 X g for 30 min and the particles were redissolved by sonicated in 3.75 mL ethanol for 20 min (at this point the solution could be stored in 4C for further use). The solution was stirred and 8.5 µL of TEOS (Sigma-Alderich) was added, followed by 40 µL of 30% ammonia. The solution was left to stir 6-10 hours after which it was purified by centrifugation for 15 min at 15000 X g and washed twice (10 min at 15000 X g) and finally suspended with 1 mL ethanol. For long-term storage, the solution was placed in Acetone instead.

S12 RAW cells

RAW-Blue™ Cells(InvivoGen), an NF-κB-SEAP reporter Cell line derived from the murine RAW 264.7 macrophages, were cultured in DMEM (DMEM, high glucose, pyruvate Gibco 11995073) supplemented with 10% (v/v) heat-inactivated fetal bovine serum (Gibco 16140071), and Antibiotic-Antimycotic (1X) from gibco (100 units/mL of penicillin, 100 µg/mL of streptomycin, and 0.025 µg/mL of Gibco Amphotericin B), with 100 µg/mL Normocin and 100 µg/mL selection antibiotic Zeocin. The cells were kept in a humidified 5% CO_2 atmosphere at 37°C.

S13 Imaging system

NV measurements were performed using a home-built confocal microscope. Optical excitation was provided by a 520 nm pulsed laser (LABS electronics DLnsec) and focused onto the sample (confocal mode) using a X60, NA=1.49 oil-immersion objective (Olympus APON60XOTIRFl). Translation of the excitation beam was done using a fast steering mirror (Newport FSM-300). Epifluorescence emission was separated from the excitation beam using a dichroic filter (Chroma T610lpxr) and filtered (Semrock LP01-594R-25) before being focused onto a single-photon counting module (Excelitas, SPCM-ARQH-14). For cell imaging, we used white light and a 488 nm (Cobolt 06-MLD 100 mW) in a wide-field mode for cells and NDs, respectively. The system was equipped with a live-cell chamber (Invivo Scientific, STEV.ECU.HC5 STAGE TOP) for ZFS measurements in live cells at 37 °C.

S14 Cellular uptake

Measurements were made after incubation of ∼12,000 cells with 15 µg/mL of core diamond nanocrystals in media (2.25 μg core diamond nanoparticles per 10,000 cells), such that the cells were exposed to a similar number of bare and core-shell particles. Wide-field fluorescence and bright field images were analyzed with an automated ImageJ script to prevent bias. Images were split by channel, and the red channel was processed by background subtraction (rolling ball radius = 50 pixels), followed by median filtering (radius = 1 pixel), Otsu thresholding, and conversion to a binary mask. Channels bleed-through was not an issue as the only fluorescent channel to be read was the red nanoparticles channel. Particle-positive resgions were identified using the Analyze Particles tool (size >2 pixels), and ROIs were stored for downstream quantification. Manually defined cell ROIs were assigned for each cell and saved in the ROI Manager. For each cell, total red fluorescence intensity (from original fluorescence image) and total internalized particle area (from binary mask) were quantified by intersecting cell ROIs with the particles mask. All results were exported per cell for statistical analysis.

S15 Lysosomal co-localization

Sample preparation

Following incubation with bare and core-shell particles, cells in each well were fixed with 100 μL 4% PFA (Image-iT™ Fixative Solution, Thermo Fisher Scientific, cat# I28800) for 15 min at RT. Following fixation, cells were washed three times with PBS and permeabilized using 100 μL per well of 0.05% saponin in PBS (Thermo Scientific Chemicals, cat# J63209.AK) for 10 min at RT. Cells were then washed twice with PBS and incubated in 5% bovine serum albumin (BSA) for 30 min to block nonspecific binding.

For lysosomal staining, cells were incubated with anti-CD107a (LAMP-1) monoclonal antibody (clone eBio1D4B, Alexa Fluor 488-conjugated, Thermo Fisher eBioscience™, cat# 53-1071-82) at a 1:200 dilution in blocking buffer for 60 min at 4 °C. After antibody incubation, cells were washed thoroughly with PBS, followed by nuclear staining using 300 nM DAPI in PBS (Pierce DAPI Nuclear Counterstain, Thermo Fisher, cat# 62248) for ∼5 min at RT, protected from light. Excess DAPI was removed by washing cells 2–3 times with PBS before imaging with confocal microscopy (SoRa Subdiffraction Marianas Spinning Disk Confocal, UChicago Integrated Light Microscopy Core).

Image analysis

Co-localization analysis between nanocrystals (red) and lysosomes (green) was conducted using an automated ImageJ [17] script to batch-process matched image pairs: red and green channels. Images were then processed manually using the JACoP plugin to remove non-cellular pixels containing red or green signal and to optimize thresholding. Manders' coefficients were extracted for each image and combined into a weighted average for each time point, with weights corresponding to the number of cells per image, as shown in Figure E6(c,d). Images were collected over several areas in 1-2 different wells for each group. The number-of-cells weighted Manders' coefficients obtained for each image were used for averaging and statistical analysis.

S16 Flow cytometry for polarization and toxicity assays

Cells were analyzed with Aurora (Cytek) spectral flow cytometer at the University of Chicago Cytometry and Antibody Technology Facility. Data was analyzed using FlowJo software (BD Biosciences). Cells were identified using forward and side scatter then single cells were selected using forward scatter area and height. Another round of single cell selection was done using side scatter area and height. Dead cells were excluded by selecting the LIVE/DEAD negative population. For viability study (Figure E8(a)), percentage of LIVE/DEAD negative population was reported as live cells. Percentage of populations positive for EdU, as well as mean fluorescence intensity (MFI) of CD86, TNFα, CD206, and CD163 were determined using their respective channels. All positive gates were set using FMO control for that fluorophore and the unstained negative control. See Figures E9 and E16 for specific gating strategy information for the EdU and the polarization assays, respectively.

S17 Biological mechanism measurements

Theoretical estimation for ZFS-dependence on pH

A basic theoretical estimation for the dependence of ZFS on pH is obtained using the Choup-Gouy-Stern theory [18], initially developed to accurately describe the correction to the electrostatic potential within electrolyte solution by also accounting for the influence of cations/ions atoms absorbed at the surface, also known as Stern layer. We consider carboxylate groups, with pKa≈ 4, as the main surface group affected by pH on the surface of oxidized bare nanocrystal. The carboxylates' surface coverage is set at 5.6%, as obtained by averaging literature values ranging from 2.3% to 9.2% [19–22]. The protonation of these groups modifies the electrical properties at the surface due to the presence of additional charges (H^+) that reduce the corresponding surface potential.

The corresponding drop in surface potential due to protonation is calculated from Poisson's equation as:

$$\phi\left(p\right) = \phi_S - \frac{ed}{\varepsilon} p\sigma, \tag{5}$$

where $\varepsilon = 5.7 * 8.8542 * 10^{-12} \; \frac{C}{V*m}$ is the electric permittivity, ϕ_S is the electrostatic potential of a fully non-protonated diamond surface (0.5 V at pH 7 [7]), $e = 1.6 * 10^{-19} \; C$ the dipole charge, $d = 1 * 10^{-10} \; m$ the dipole displacement, σ the areal density of carboxylate complex and p the percentage of protonated complex. Using the density of carbon atoms at [100] surface as $2/(3.5\,\text{Å})^2$, we can now calculate the change in surface potential in response to protonation of surface groups. We next use the Henderson-Hasselbalch equation to calculate the fraction of protonated carboxylates when changing the pH from 7 (0%) to 4.5 (24%), corresponding to the pH changes expected during the acidification of lysosomes [23, 24]. Putting all together, we obtain a surface potential change from 0.5 V at pH 7 to 0.4303 V at pH 4.5. Figure E1(c) can be used to assess the change in ZFS in response to this decrease in surface potential, revealing a change ranging from 0.4 to 33 kHz for the lowest and highest value of d', respectively. These values are at or below our detection limit and are negligible compared to the ZFS changes we recorded. We next confirm this concept experimentally.

ZFS in varying pH

Solutions of varying pH were prepared by subsequent dilution of 1 mM HCl before ~200 seconds of ZFS tracking data were taken for bare nanocrystals (n = 4) incubated in pH 7, 6, 5, 4.5, 4, and 3.5, to fully represent even

extreme cases of subcellular acidification. Between each measurement, the sample was washed three times with water, and then a subsequent two times with the required concentration solution. ZFS tracking data displayed roughly Gaussian statistics, and so for each pH value, an average ZFS and standard deviation could be compiled. One data point (nanocrystal 3 at pH = 4) represents a statistical outlier, as determined by Tukey's Method, but was included in the significance analysis for completeness. The average ZFS values measured across all other combinations of pH values and diamond nanocrystals suggest no ZFS dependence on pH variations in the range 7 to 3.5. Results are shown in Figure E13(a).

ZFS in BSA

A solution of 300 g/L BSA (Sigma Aldrich) in water was used to represent protein concentration in living cells [25]. Data was taken for bare nanocrystals ($n = 6$) incubated in water before the BSA solution was added, and the ZFS was tracked again for the same nanocrystals. We note that the nanocrystal sample prepared for BSA measurements had a larger density than normal. As such, in order to ensure the continuous optimization of the diamond nanocrystal fluorescence results in focusing on a nearby nanocrystal, we implemented a fluorescence intensity and localization filter, removing rare events of grouped data points that showed abnormal spatial displacement and fluorescence intensity jumps. Therefore, the total time for each measurement varied slightly. The average ZFS values measured across the 6 nanocrystals reveal that most exhibited shifts towards lower frequencies that were small compared to those measured in inflamed cells and were not statistically significant ($p = 0.613$) compared to measurements in water, which were taken as the reference ZFS. Results are shown in Figure E13(b).

ZFS in varying H_2O_2 concentration

Solutions of varying H_2O_2 concentrations were prepared by subsequent dilution of 30% H_2O_2 stock solution to a range of 0-500 µM, as to represent varying ROS concentration in resting and LPS-stimulated RAW cells [26, 27]. Data was taken for bare nanocrystals ($n = 3$) incubated in water before the H_2O_2 solutions were added, and the ZFS was tracked again for the same nanocrystals. The average ZFS values measured across the 3 nanocrystals revealed a concentration-dependent shift that approached significance for 0-100 µM. The average ZFS shift for nanocrystals incubated in 500 µM exhibited significant ($p = 0.048$), yet moderate shift of 156±43 kHz. Results are shown in Figure E13(d).

ZFS in lysate of LPS-stimulated cells

10 million RAW 264.7 cells were incubated at 37 °C for 1.5 hours with 6 mL of 1.5 µg/mL LPS in complete media to produce lysate concentration of 1.67e6 cell equivalents per mL media and minimize dilution of the soluble cellular components. Following incubation, the media was extracted and cells were lysed with 6 mL of 0.05% saponin in media for 10 min on ice. Cells were scraped to detach membranes, and the lysate was applied directly to the particles being measured on our custom-built microscope. The lysate was not frozen and applied fresh after equilibrating to room temperature. Data was taken for bare nanocrystals ($n = 4$) incubated in water before the lysate was added, and the ZFS was tracked again twice for each nanocrystal. The average ZFS values measured across the 3 nanocrystals revealed significant negative ZFS shifts of 268±64 kHz ($p = 0.019$) and 323±78 kHz ($p = 0.018$) for the first and second measurement in lysate, respectively. A subsequent measurement in water showed partial recovery of the ZFS to a level not significantly different from the initial measurement in water ($p = 0.250$).We note that no ROS scavengers or other rescue assays were used in our work.

The measurement was repeated using core-shell particles ($n = 3$). Following measurements in water, new lysate from freshly stimulated cells was added, and the ZFS was tracked again for the same particles that were measured in water. The average ZFS values measured across the 3 particles revealed no systematic ZFS shifts ($p = 0.243$), although random ZFS changes were seen in 2 of the 3 particles. A subsequent rinse and measurement in water again did not produce significant changes ($p = 0.239$). Results are shown in Figure E13(c).

Scope of the preliminary mechanistic investigation

The experiments in Figure E13 were intended as a preliminary test of potential interactions between diamond nanocrystals and selected biological components. Lysate from inflamed cells and H_2O_2 produced ZFS shifts com-

parable to those observed in live inflamed macrophages, whereas BSA and physiological pH changes did not. These results support the physical plausibility of our band-bending model but were not designed to resolve the detailed biochemical mechanisms at the nanoparticle–cell interface. A systematic analysis of surface terminations, protein coronas, and oxidative species will be required to fully delineate these effects and is the subject of ongoing work.

S18 Statistics

Allan Variance Analysis

To compute Allan variances for individual NV ZFS time-series data, we recorded the raw fluorescence data for I_1 and I_2 and artificially generated data sets for measurements with larger averaging time by summing over subsequent fluorescence data. For example, if our initial data averaged fluorescent data measurements for 1 second, then we would generate artificial data for 2 second of measurement by summing the I_1 and I_2 data from both measurements, respectively. Using the fluorescence data for each averaging time, we generated time-series data of the ZFS in the process described in Sec. S3, and could then compute the $\tau = 0$ Allan variance for each dataset. Piecing these values together, the τ axis is the averaging time and the Allan variance axis is the starting Allan variance value for each averaging time.

To compute the average Allan variance plot for the time-series ZFS data of bare (n = 8) and core-shell (n = 12) nanodiamonds in PBS, we applied a weighted average of the individual Allan variance plots with the weights being the total measurement duration. This method ensures that the aggregate plot accurately reflects the contribution of each time series based on its respective measurement period. We then obtained Allan variance values of 6.90 for the bare nanodiamonds and 4.59 for the core-shell nanodiamonds. The minimum Allan variance recorded was 1.70 for the bare nanodiamonds and trended below 0.82 for the core-shell nanodiamonds, though the average Allan variance plot for core-shell did not exhibit a clear minimum point. These findings suggest that in PBS, the core-shell diamond nanocrystals ZFS is more stable against both low and high-frequency noise sources than the bare diamond nanocrystals ZFS.

Augmented Dickey-Fuller Test

The Augmented Dickey-Fuller (ADF) Test is a statistical test used to determine whether a given time series is stationary or contains a unit root, which would indicate non-stationarity. It includes lagged differences of the time series to account for autocorrelation in the data. The ADF test estimates the following regression equation:

$$\Delta y_t = \alpha + \gamma y_{t-1} + \sum_{i=1}^{p} \delta_i \Delta y_{t-i} + \epsilon_t$$

Where:

- $\Delta y_t = y_t - y_{t-1}$: The first difference of the time series, capturing the change in values.
- α: Constant or intercept term
- γy_{t-1}: Coefficient on the lagged level of the series, which is tested for significance.
- $\sum_{i=1}^{p} \delta_i \Delta y_{t-i}$: Lagged differences of the series, included to control for autocorrelation.
- ϵ_t: White noise error term.

The key test statistic is associated with γ, which indicates the presence of a unit root. The null hypothesis (H_0) is that the series has a unit root ($\gamma = 0$), meaning it is non-stationary. The alternative hypothesis (H_1) is that the series does not have a unit root ($\gamma < 0$), meaning it is stationary.

In the ADF test, the inclusion of lagged differences (Δy_{t-i}) is critical for addressing autocorrelation in the series. However, choosing the right number of lags (p) is non-trivial. To automate this process, the ADF test can use the Akaike Information Criterion (AIC), which evaluates models with different lag lengths and selects the one that minimizes the AIC value.

The AIC is defined as:

$$\mathrm{AIC} = 2k - 2\ln(L),$$

where:

- k: Number of estimated parameters (including the intercept and lag terms).
- $\ln(L)$: Log-likelihood of the model.

Using AIC ensures a balance between model fit and complexity, selecting the optimal lag length to avoid overfitting or underfitting.

We use the *statsmodels.tsa.stattools.adfuller* Python package to conduct the ADF test on our time-series data, and implement a threshold of a p-value < 0.05 to indicate the rejection of H_0, suggesting the series is stationary. A p-value of 0.557 indicates that the ZFS time series for bare nanocrystals in PBS is a non-stationary process, whereas, for core-shell particles, a p-value of 3.06×10^{-4} suggests a stationary process.

References

[1] F. Dolde, H. Fedder, M. W. Doherty, T. Nöbauer, F. Rempp, G. Balasubramanian, T. Wolf, F. Reinhard, L. C. L. Hollenberg, F. Jelezko, J. Wrachtrup. *Nat. Phys.* **2011**, *7*, 6 459.

[2] J. Michl, J. Steiner, A. Denisenko, A. Bülau, A. Zimmermann, K. Nakamura, H. Sumiya, S. Onoda, P. Neumann, J. Isoya, J. Wrachtrup. *Nano Lett.* **2019**, *19*, 8 4904.

[3] D. R. Candido, M. E. Flatté. *Phys. Rev. B* **2024**, *110* 024419.

[4] M. W. Doherty, F. Dolde, H. Fedder, F. Jelezko, J. Wrachtrup, N. B. Manson, L. C. L. Hollenberg. *Phys. Rev. B* **2012**, *85* 205203.

[5] Policies & Practices, P. Conduct **2018**.

[6] H. Y. Chen, S. A. Bhave, G. D. Fuchs. *Phys. Rev. Appl.* **2020**, *13*, 5.

[7] U. Zvi, D. R. Candido, A. M. Weiss, A. R. Jones, L. Chen, I. Golovina, X. Yu, S. Wang, D. V. Talapin, M. E. Flatté, A. P. Esser-Kahn, P. C. Maurer. *Proc. Natl. Acad. Sci. U. S. A.* **2025**, *122*, 21 e2422542122.

[8] P. Deák, B. Aradi, M. Kaviani, T. Frauenheim, A. Gali. *Phys. Rev. B Condens. Matter* **2014**, *89*, 7 075203.

[9] O. A. Shenderova, A. I. Shames, N. A. Nunn, M. D. Torelli, I. Vlasov, A. Zaitsev. *J. Vac. Sci. Technol. B Nanotechnol. Microelectron.* **2019**, *37*, 3 030802.

[10] S. K. R. Singam, M. Nesladek, E. Goovaerts. *Nanotechnology* **2019**, *31*, 10 105501.

[11] G. Baffou, H. Rigneault, D. Marguet, R. Jaffiol. *Nature Methods* **2014**, *11*, 9 899.

[12] C. Loesberg, J. van Miltenburg, R. van Wuk. *Journal of Thermal Biology* **1982**, *7*, 4 209.

[13] N. C. Catalog. CellROX® oxidative stress reagents.

[14] K. Shang-Guan, M. Wang, N. M. P. S. Htwe, P. Li, Y. Li, F. Qi, D. Zhang, M. Cao, C. Kim, H. Weng, H. Cen, I. M. Black, P. Azadi, R. W. Carlson, G. Stacey, Y. Liang. *Plant Physiol.* **2018**, *176*, 3 2543.

[15] H.-Y. Hsu, M.-H. Wen. *J. Biol. Chem.* **2002**, *277*, 25 22131.

[16] W. Stöber, A. Fink, E. Bohn. *J. Colloid Interface Sci.* **1968**, *26*, 1 62.

[17] C. A. Schneider, W. S. Rasband, K. W. Eliceiri. *Nat. Methods* **2012**, *9*, 7 671.

[18] K. B. Oldham. *Journal of Electroanalytical Chemistry* **2008**, *613*, 2 131.

[19] A. Wolcott, T. Schiros, M. E. Trusheim, E. H. Chen, D. Nordlund, R. E. Diaz, O. Gaathon, D. Englund, J. S. Owen. *J. Phys. Chem. C Nanomater. Interfaces* **2014**, *118*, 46 26695.

[20] C. Marchal, L. Saoudi, H. A. Girard, V. Keller, J.-C. Arnault. *Adv. Energy Sustain. Res.* **2024**, *5*, 3 2300260.

[21] R. Zulkharnay, G. Zulpukarova, P. W. May. *Appl. Surf. Sci.* **2024**, *658*, 159776 159776.

[22] X. Wang, A. R. Ruslinda, Y. Ishiyama, Y. Ishii, H. Kawarada. *Diamond and Related Materials* **2011**, *20*, 10 1319.

[23] J. M. Blander, R. Medzhitov. *Nat. Immunol.* **2006**, *7*, 10 1029.

[24] D. G. Russell, B. C. Vanderven, S. Glennie, H. Mwandumba, R. S. Heyderman. *Nat. Rev. Immunol.* **2009**, *9*, 8 594.

[25] K. R. Albe, M. H. Butler, B. E. Wright. *J. Theor. Biol.* **1990**, *143*, 2 163.

[26] L. M. Paardekooper, I. Dingjan, P. T. A. Linders, A. H. J. Staal, S. M. Cristescu, W. C. E. P. Verberk, G. van den Bogaart. *Front. Immunol.* **2019**, *10* 1216.

[27] H. Wang, X. Xu, Z. Yin, M. Wang, B. Wang, C. Ma, J. Wang, W. Kang. *Int. J. Immunopathol. Pharmacol.* **2021**, *35* 20587384211010058.
